# Comprehensive analysis of circular RNA expression dynamics and competitive endogenous RNA network mechanisms during postnatal liver development in juvenile goats

**DOI:** 10.5713/ab.250689

**Published:** 2025-11-25

**Authors:** Qing Li, Shanfeng Du, Jianmin Wang, Jiaqing Hu, Tianle Chao

**Affiliations:** 1 Shandong Provincial Key Laboratory of Animal Biotechnology and Disease Control and Prevention, College of Animal Science and Veterinary Medicine, Shandong Agricultural University, Tai’an City, China; 2 Key Laboratory of Efficient Utilization of Non-grain Feed Resources (Co-construction by Ministry and Province), Ministry of Agriculture and Rural Affairs, Shandong Agricultural University, Tai’an City, China; 3 College of Agricultural Science and Technology, Shandong Agriculture and Engineering University, Jinan, China

**Keywords:** Circular RNA, Competitive Endogenous RNA Network, Laiwu Black Goat, Lipid Metabolism, Liver Development

## Abstract

**Objective:**

This study analyzes the dynamic expression profile and functional mechanisms of circular RNA (circRNA) in the liver of young goats during development from birth to the early weaning stage.

**Methods:**

This study performed transcriptome sequencing on liver tissues from Laiwu Black goats at five key developmental stages after birth (1 day, and 2, 4, 8, and 12 weeks).

**Results:**

A total of 178 differentially expressed circRNAs were identified across the five developmental stages. Functional enrichment analysis showed that the source genes of these circRNAs are involved in key pathways such as immune response, lipid metabolism, and cell signaling. Persistently upregulated circRNAs (cluster 9) are related to tryptophan metabolism, potentially influencing the inflammatory response and energy homeostasis during weaning stress in young goats. In contrast, persistently downregulated circRNAs (cluster 0) may promote the change in liver metabolism from glucose to fatty acid oxidation by relieving inhibition on pathways such as AMPK and insulin signaling pathway. Based on the competitive endogenous RNA (ceRNA) mechanism, the study constructed a regulatory network consisting of 20 circRNAs, 15 microRNAs, and 21 mRNAs. Specifically chi-miR-532-3p/*CYP8B1*/circ101504503815046736, chi-miR-542-5p/*ACACB*/circ10_ 21217626_21219471, and miR-542-5p/*ACACB*/circ10-21217626-21219471 were identified as potential key ceRNA axes. Furthermore, this study confirmed the regulatory relationship among chi-miR-532-3p, *CYP8B1* and circ10-21217626-21219471 through dual luciferase reporter gene experiments.

**Conclusion:**

This study reveals the dynamic expression profile of circRNA during goat liver development, suggesting its potential role in regulating metabolic and immune pathways through the ceRNA mechanism. This offers a novel perspective on post-transcriptional regulation in ruminant in liver development.

## INTRODUCTION

The liver is the metabolic hub of mammals, responsible for coordinating key physiological processes such as glycogenesis, lipid metabolism, detoxification, and immune homeostasis. [1,2]. The realization of its functions depends on the dynamic expression regulation of genes among highly heterogeneous cells [3]. This regulatory network is particularly critical during early development, where, perinatally, the liver experiences a metabolic reshaping from a glucose-consuming organ to one for gluconeogenesis and storage, involving the coordinated changes of key factors regulating glycolysis and gluconeogenesis [4].

In recent years, circular RNAs (circRNAs), a type of covalently closed single-stranded RNA molecule formed from mRNA precursors through back-splicing, have been confirmed as a new class of important molecules in gene regulatory networks due to their stability and tissue-specific expression patterns [5]. CircRNAs can finely regulate gene expression at the post-transcriptional level through various mechanisms, including acting as microRNA (miRNA) sponges, interacting with RNA-binding proteins, or participating in translation [6]. The competitive endogenous RNA (ceRNA) mechanism, in which circRNAs bind with miRNAs through their miRNA response elements (MREs) to relieve miRNA repression on their target genes, thereby indirectly regulating mRNA stability and translation efficiency, is particularly noteworthy [7].

Although preliminary reports exist regarding the functions of circRNAs in the skeletal muscle, fat, and ovaries of goats [8–10], their dynamic expression profile and potential regulatory roles in this core metabolic organ, the liver, during the postnatal development process remain unknown. Therefore, we hypothesize that the expression profile of circRNAs in the early development of goat liver exhibits significant temporal dynamics and that these differentially expressed circRNAs (DECs) may serve as important post-transcriptional regulatory elements involved in regulating the core gene network for the maturation of liver metabolic functions.

Based on this, this study constructs a temporal expression profile of goat liver circRNAs by conducting transcriptome sequencing of Laiwu Black goat liver tissues at five key time points: postnatal day 1 (D1), 2 weeks (W2), 4 weeks (W4), 8 weeks (W8), and 12 weeks (W12). By integrating differential expression analysis, ceRNA network construction, functional enrichment analysis, and molecular experimental validation, the aim is to reveal the molecular basis by which circRNAs regulate liver metabolism and immune homeostasis through the ceRNA mechanism. This research provides a new theoretical basis for understanding the post-transcriptional regulatory mechanisms of early liver development in ruminants.

## MATERIALS AND METHODS

### Experimental animals and sample collection

A total of 25 healthy female Laiwu Black goats were selected from the Laiwu Black Goat Original Breeding Farm (Shandong Fengxiang Animal Husbandry Seed Industry Technology). The animals were divided into five age groups with five biological replicates per group: 1 day (D1), 2 weeks (W2), 4 weeks (W4), 8 weeks (W8), and 12 weeks (W12). There is no genetic relationship among the experimental goats. Within the first 30 days after birth, the goat kids were kept with their dams and allowed to suckle freely. From day 30 to 60, solid concentrate feed was gradually introduced as a supplement. After 60 days of age, the animals were completely weaned and switched to a solid feed–only diet. All goats were raised under a standardized rearing protocol with ad libitum access to feed and water. On the same day, the five developmental stages of goats were euthanized, and liver tissue samples were immediately collected and rapidly frozen in liquid nitrogen for subsequent RNA sequencing.

### Total RNA extraction and quality control

Total RNA was extracted from liver tissue samples using TRIzol reagent (Invitrogen) according to the manufacturer’s instructions. To remove genomic DNA contamination, the RNA samples were treated with RNase-free DNase I (TaKara). RNA concentration and purity were assessed using a NanoDrop 2000 spectrophotometer (NanoDrop Technologies). RNA integrity was evaluated using an Agilent 2100 Bioanalyzer (Agilent Technologies). All samples had RNA integrity numbers (RIN) greater than 8.0, meeting the quality standards for library construction and sequencing.

### Strand-Specific library construction and transcriptome sequencing

Ribosomal RNA (rRNA) was removed using the Ribo-Zero Magnetic Kit (EpiCentre). Strand-specific sequencing libraries were then prepared following the instructions of the Illumina TruSeq Stranded Total RNA Library Prep Kit. After RNA fragmentation, first-strand cDNA was synthesized using random hexamer primers. During second-strand synthesis, dUTP was incorporated to preserve strand orientation. The resulting cDNA was purified, end-repaired, adenylated, and ligated with indexed adapters. cDNA fragments of 200–300 bp were enriched by polymerase chain reaction (PCR) amplification (15 cycles). The final libraries were quality-checked on an Agilent 2100 Bioanalyzer. Qualified libraries were quantified and normalized, and paired-end 150 bp (PE150) sequencing was performed on an Illumina NovaSeq 6000 platform. The datasets supporting the conclusions of this article are available in the NCBI database. reference number: GSE194190 and PRJNA770352.

### Sequencing data processing, alignment, and circular RNA identification

Raw sequencing data first underwent stringent quality control and adapter trimming using fastp [11]. Further preprocessing was performed with SeqPrep (v1.2, https://github.com/jstjohn/SeqPrep) and Sickle (v1.33, https://github.com/najoshi/sickle) to eliminate low-quality reads according to the following criteria: (1) adapter removal; (2) 3′ trimming (Q<20), with reads discarded if any base remained with Q<10; (3) exclusion of reads with excessive Ns; (4) retention of sequences >30 bp. Subsequently, high-quality clean reads were aligned to the goat reference genome (ARS1 (GCA_001704415.1)) using HISAT2 [12]. Transcript assembly was performed with StringTie [13]. On this basis, circRNA identification was further carried out based on back-splice junction (BSJ) reads. This involved using CIRI [14] to analyze CIGAR values in SAM files to scan for paired cross-junction signals, then filtering junction reads based on paired-end mapping and the GT-AG splicing signal. Finally, we applied the deletion-merge algorithm to detect imbalanced junction reads, which helped suppress false positives caused by homologous gene similarity and repetitive sequences. To correct for variations in sequencing depth and enable direct cross-sample comparison, circRNA expression levels were normalized using the Reads Per Million (RPM) method [15].

### Differential expression analysis and functional annotation

Differential expression analysis of circRNAs was performed using DESeq2 [16]. DECs were identified with the thresholds of p.adj<0.05 and |log2FoldChange|≥1. Short Time-series Expression Miner (STEM) was used to analyze the expression patterns of the identified DECs [17]. To explore the potential functions of the DECs, Gene Ontology (GO) enrichment and Kyoto Encyclopedia of Genes and Genomes (KEGG) pathway analyses were conducted on their host genes. GO enrichment was performed using clusterProfiler [18], and KEGG pathway analysis was carried out with KOBAS [19]. A p-value<0.05 was considered statistically significant.

### Construction of the competitive endogenous RNA regulatory network

To investigate the potential regulatory mechanisms of circRNAs as ceRNAs, we integrated concurrently obtained mRNA and miRNA expression profiles [20,21]. Binding sites between DECs and differentially expressed miRNAs (DEMs) were predicted using miRanda [22] and RNAhybrid [23]. Expression correlation (Pearson correlation coefficient) was calculated between DECs and differentially expressed mRNAs (DEGs). Pairs showing significant negative correlation (Spearman’s correlation coefficient [SCC]<–0.50, p.adj<0.05) between circRNA/mRNA and miRNA were selected. Pearson correlation was further calculated between DEMs in the ceRNA network and phenotypic traits (body weight and liver weight) of the goats (Supplement 1). The circRNA–miRNA–mRNA regulatory interactions were visualized using Cytoscape (v3.9.1) and Origin (v9.95).

### Quantitative real-time polymerase chain reaction validation

To validate the reliability of the high-throughput sequencing results, two DECs and two non-DECs were selected for quantitative real-time PCR (qRT-PCR). Total RNA was extracted from liver tissues using TRIzol reagent. cDNA synthesis for circRNAs and mRNAs was performed using the Evo M-MLV Reverse Transcription Kit II (Accurate Biotechnology), which includes a gDNA removal step. For miRNA, reverse transcription was carried out using the miRNA First Strand cDNA Synthesis Kit (Accurate Biotechnology) according to the manufacturer’s instructions.

In the qPCR assays, β-actin was used as the internal reference gene for circRNAs and mRNAs [24], and U6 was used for miRNA. Amplification was performed on a LightCycler 96 Real-Time PCR System (Roche) using SYBR Green Pro Taq HS Premix (Accurate Biotechnology). The reaction protocol consisted of an initial denaturation at 95°C for 30 s, followed by 40 cycles of denaturation at 95°C for 5 s and annealing/extension at 60°C for 30 s. All primer sequences are listed in Supplement 2. The relative expression levels were calculated using the 2^−ΔΔCt^ method, with three technical replicates per sample.

### Dual-Luciferase reporter assay

Based on RNAhybrid predictions, wild-type (WT) and mutant luciferase reporter vectors were constructed. The putative binding region for chi-miR-532-3p in the 3′UTR of *CYP8B1* was cloned into the pmirGLO vector to generate the WT reporter (CYP8B1-WT). The mutant reporter (CYP8B1-MUT) was generated by site-directed mutagenesis of the predicted binding sites. The same procedure was applied to the complementary region of chi-miR-532-3p in circ 10_15045038_ 15046736 (circ10) to generate circ10-WT and circ10-MUT vectors. These vectors were co-transfected with miR-532-3p mimic or negative control mimic (NC) into 293T cells using Lipofectamine 3000 reagent according to the manufacturer’s instructions. The cells were harvested 48 hours post-transfection, and luciferase activity was measured using a dual-luciferase reporter assay system. Renilla luciferase activity was used for normalization. Experiments were performed with three replicates.

### Statistical analysis

All data are presented as mean±standard deviation. Statistical analysis was performed using SPSS 17.0 software. Comparisons between two groups were conducted using Student’s t-test, and comparisons among multiple groups were performed using one-way analysis of variance (ANOVA). A p-value<0.05 was considered statistically significant.

## RESULTS

### Overview of circular RNA sequencing data

To characterize the expression profile of circRNAs in the liver of young goats, a total of 25 liver samples were collected from Laiwu Black goats at five postnatal developmental stages (D1, W2, W4, W8, and W12) for circRNA sequencing. After quality control using fastp (removing adapter sequences and low-quality reads), approximately 3.024 billion high-quality clean reads were retained (Q20>97.43%, Q30>92.52%) (Supplement 3). The filtered reads were aligned to the goat reference genome (Capra_hircus) using HISAT2, achieving an overall alignment rate exceeding 96% and a unique mapping rate over 85% (Supplement 3).

### Identification and differential expression analysis of circular RNAs

**Figure 1:**
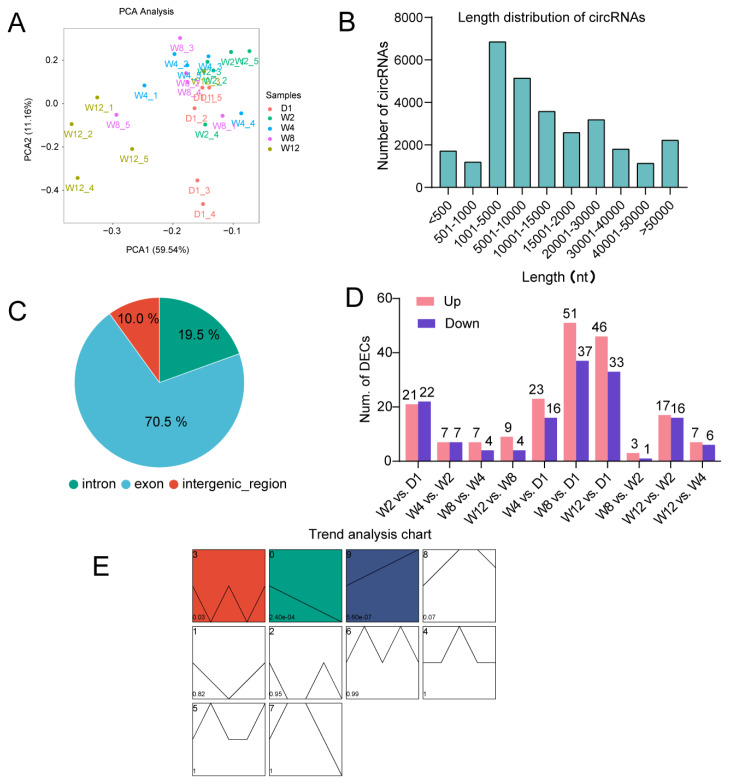
Identification and expression characterization of circular RNAs (circRNAs) during liver development in young goats. (A) Principal component analysis (PCA) of samples based on circRNA expression profiles. (B) Length distribution frequency of identified circRNAs. (C) Classification of circRNAs by genomic origin, showing the proportions of exonic, intronic, and intergenic circRNAs. (D) Number of differentially expressed circRNAs (DECs) identified in pairwise comparisons across different developmental stages. (E) Temporal expression pattern analysis illustrating the expression trends of DECs. The numbers in the upper-left corner represent the sequence names of each expression cluster, and the numbers in the lower-left corner indicate the p-values.

A total of 29,428 circRNAs were identified in liver tissues throughout early postnatal development in goats. Principal component analysis (PCA) indicated that the samples across different groups exhibited a distribution pattern closely related to developmental stages. In the early developmental stages (D1, W2, W4, W8), particularly during the W2–W8 period, the samples among the groups were not completely separated, whereas the W12 group samples were clearly separated from other groups (Figure 1A). These results suggest that the expression profile of circRNAs in goat liver shows significant stage specificity during early postnatal development. Length distribution analysis showed that the vast majority of circRNAs were shorter than 10,000 bp (Figure 1B). Composition analysis further demonstrated that exon-derived circRNAs constituted the largest proportion (70.53%, 20,756), followed by intronic circRNAs (19.50%, 5,737) and intergenic circRNAs (9.97%, 2,935) (Figure 1C). This distribution pattern is consistent with the characteristics of circRNAs found in the ruminant liver.

Through pairwise comparisons of circRNAs in goat liver across five developmental stages (D1, W2, W4, W8, W12), a total of 178 DECs (Supplement 4) were identified. Inter-group comparisons revealed that the highest number of DECs occurred between D1 and W8 (88 in total, including 51 upregulated and 37 downregulated), suggesting that the transition from the neonatal to the weaning period represents the most significant changes in liver circRNA expression profiles. In contrast, the fewest DECs were observed between W2 and W8 (only 4, including 3 upregulated and 1 downregulated), which is consistent with the PCA results, indicating that circRNA expression in goat livers during the early growth stage dominated by suckling is relatively stable. Furthermore, the number of DECs between consecutive time points significantly decreased over developmental progression, with the highest number between D1 and W2 (Figure 1D). These results suggest that these circRNAs may play important roles during the critical metabolic transition period of early liver maturation.

### Expression patterns of differentially expressed circular RNA and functional enrichment analysis of their host genes

**Figure 2:**
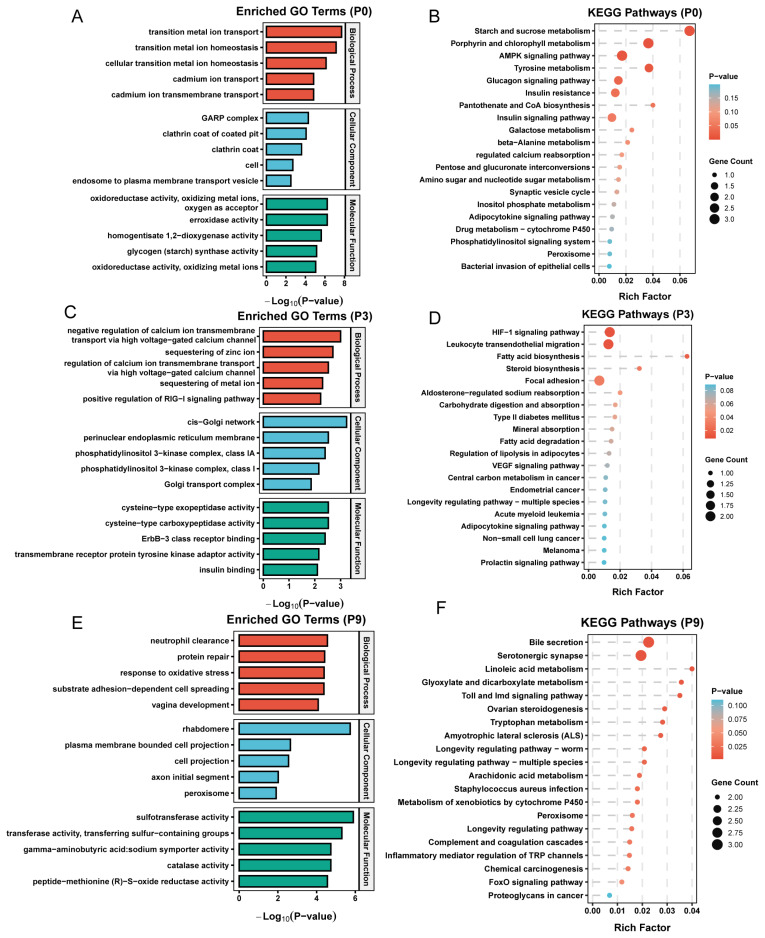
Functional enrichment analysis of source genes of differentially expressed circular RNAs (DECs) with distinct expression patterns. (A) Gene Ontology (GO) enrichment analysis was performed on the host genes of DECs derived from cluster P0. Terms are categorized into biological process (BP), cellular component (CC), and molecular function (MF). (B) Kyoto Encyclopedia of Genes and Genomes (KEGG) pathway enrichment analysis of source genes of DECs from cluster P0. (C) GO enrichment analysis was performed on the host genes of DECs derived from cluster P3. (D) KEGG pathway enrichment analysis of source genes of DECs from cluster P3. (E) GO enrichment analysis was performed on the host genes of DECs derived from cluster P9. (F) KEGG pathway enrichment analysis of source genes of DECs from cluster P9.

To investigate the role of DECs in the liver development of young goats, this study identified three clusters of DECs (P0, P3, and P9) with significant temporal expression patterns (p<0.05) through STEM cluster analysis (Figure 1E) and performed functional enrichment analysis. P0 contains 25 DECs that are consistently downregulated during postnatal development. The host genes of these DECs are significantly enriched in GO terms such as transition metal ion transport, peroxidase activity, and glycogen (starch) synthase activity (p<0.05) (Figure 2A). These genes also participate in metabolic pathways including starch and sucrose metabolism, AMPK, insulin, and glucagon signaling pathways (p<0.05) (Figure 2B). This suggests that DECs in P0 may establish a mature energy metabolism homeostasis by adapting to the nutritional transition from maternal milk to solid feed after birth. P3 contains 23 DECs that exhibit a wave-like expression pattern with high expression at D1, W4, and W12, and low expression at W2 and W8. Host genes in this cluster are significantly enriched in GO terms such as regulation of voltage-gated calcium ion channels, positive regulation of the RIG-I signaling pathway, and insulin-like growth factor receptor signaling pathway (p<0.05) (Figure 2C). Although the KEGG analysis was not statistically significant, host genes in this cluster showed enrichment trends in pathways including mTOR signaling, apoptosis, estrogen signaling, phospholipase D, and AMPK signaling (Figure 2D), suggesting that DECs in P3 may be involved in signal transduction and immune response processes, ensuring functional transition at critical points of liver development. P9 contains 31 DECs showing consistently upregulated expression. Host genes in this cluster are significantly enriched in immune-related GO terms such as neutrophil clearance, complement activation, antibiotic catabolic processes, cell-matrix adhesion, and positive regulation of hepatic stellate cell activation (p<0.05) (Figure 2E), and show significant enrichment in metabolic pathways including tryptophan metabolism, ovarian steroidogenesis, linoleic acid metabolism, serotonergic synapse, FoxO signaling pathway, and bile secretion (p<0.05) (Figure 2F). This indicates that DECs in P9 may be involved in hepatic metabolic and immune activation processes, reflecting the progressive maturation of physiological status.

### Experimental validation of circular RNA sequencing data

**Figure 3:**
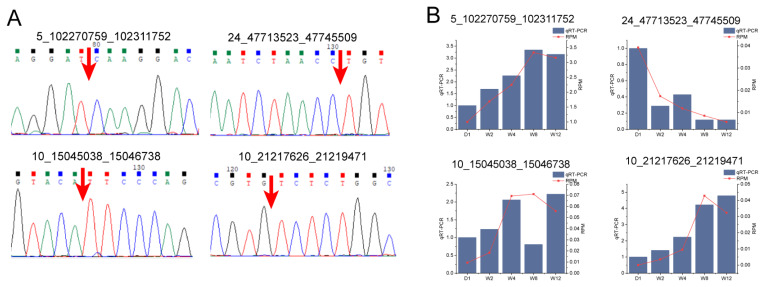
Structural and quantitative validation of circular RNAs (circRNAs). (A) Validation of back-splice junction sites of circRNAs by Sanger sequencing. The red arrows indicate the back-splice junction sites of the circRNAs. (B) Quantitative validation of circRNA expression levels by quantitative real-time polymerase chain reaction (qRT-PCR). Differentially expressed circRNAs (DECs): 10_15045038_15046738 and 10_21217626_21219471. Non-DECs: 5_102270759_102311752 and 24_47713523_47745509. D1, W2, W4, W8, and W12 denote postnatal day 1, and weeks 2, 4, 8, and 12, respectively.

To experimentally validate the reliability of the circRNA sequencing data, four circRNAs were selected for qPCR analysis, comprising two DECs (10_15045038_15046738 and 10_21217626_21219471) and two non-DECs (5_102270759_ 102311752 and 24_47713523_47745509). PCR amplification followed by Sanger sequencing was performed to confirm the BSJs of these circRNAs. The results demonstrated that the back-splice sites were fully consistent with the circularization sites predicted from the transcriptome sequencing data (Figure 3A). Subsequently, the expression dynamics of these four circRNAs across the five developmental time points were examined using qRT-PCR. The expression trends observed were highly consistent with the high-throughput sequencing data (Figure 3B). Collectively, these experiments confirm that the circRNA identification and expression profiling data obtained in this study are accurate and reliable.

### Construction of key competitive endogenous RNA network and identification of core regulatory axes

To further elucidate the functions of DECs during liver development, a DECs–DEMs–DEGs regulatory network was constructed based on the ceRNA hypothesis by integrating miRNA and mRNA expression data from our previous transcriptomic studies (Supplement 5). KEGG pathway enrichment analysis of the DEGs within the network revealed significant enrichment in multiple biological processes closely related to liver function (p<0.05), the intestinal immune network for IgA production, steroid hormone biosynthesis, fatty acid biosynthesis, Th17 cell differentiation, and the adipocytokine, calcium, and PI3K-Akt signaling pathways (Supplement 6).

**Figure 4:**
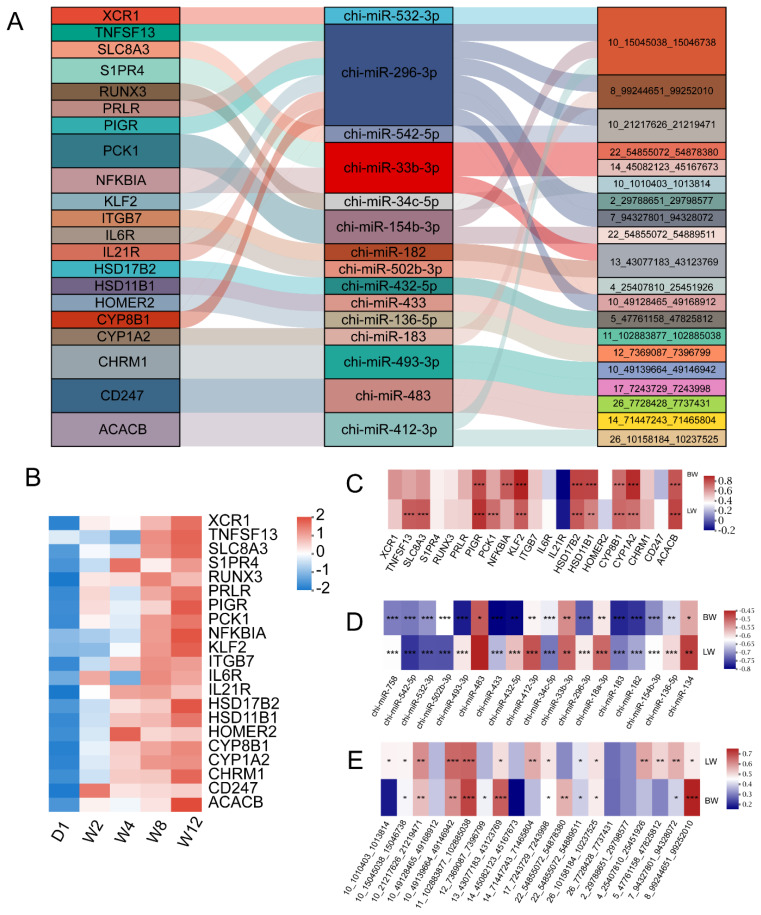
Construction and analysis of the competitive endogenous RNA (ceRNA) regulatory network during postnatal liver development in young goats. (A) Network diagram of key differentially expressed circular RNAs (circRNAs; DECs), differentially expressed microRNA (miRNAs; DEMs), differentially expressed genes (DEGs) based on the ceRNA mechanism. Nodes represent DEGs (left), DEMs (middle), and DECs (right), and edges represent predicted ceRNA interactions. (B) Heatmap showing the expression patterns of DEGs in the key ceRNA network across five developmental stages. (C–E) Heatmap of Spearman correlations between the expression levels of differentially expressed genes, miRNAs, and circRNAs in the ceRNA network and body or liver weight. D1, W2, W4, W8, and W12 denote postnatal day 1, and weeks 2, 4, 8, and 12, respectively. * p<0.05, ** p<0.01, *** p<0.001.

Based on the above pathway information, we extracted a key ceRNA subnetwork implicated in immune regulation, metabolic processes, and signal transduction. This subnetwork consists of 20 DECs, 15 DEMs, and 21 DEGs (Figure 4A). Among these components, circRNA 10_15045038_ 15046738 shows connectivity to the largest number of DEMs, while miR-296-3p targets the highest number of DECs. Figure 4B displays the expression patterns of DEGs within the ceRNA network across developmental stages, revealing that most genes maintain elevated expression levels from W4 to W12. Correlation analysis between DEGs, DEMs, and DECs in the Cerna network and growth traits (Figures 4C–4E) further demonstrated that ATP-binding cassette sub-family B member 11 (*ABCB11*), acetyl-CoA carboxylase beta (*ACACB*), cytochrome P450 family 8 subfamily B member 1 (*CYP8B1*), cytochrome P450 family 1 subfamily a member 2 (*CYP1A2*), hydroxysteroid 11-beta dehydrogenase 1 (*HSD11B1*), hydroxysteroid 17-beta dehydrogenase 2 (*HSD17B2*), KLF transcription factor 2 (*KLF2*), and polymeric immunoglobulin receptor (*PIGR*) all show significant positive correlations with both body weight (BW) and liver weight (LW). In contrast, most DEMs, such as chi-miR-532-3p and chi-miR-542-5p, exhibited significant negative correlations with BW and LW (p<0.05); DECs, such as 10_15045038_15046736 and 10_21217626_21219471, showed significant positive correlations with BW and LW (p<0.05). Based on these results, we identified several key ceRNA regulatory axes, including chi-miR-532-3p/circ10_15045038_15046736/*CYP8B1*, chi-miR-296-3p/*KLF2*/circ10_15045038_15046736, and miR-542-5p/*ACACB*/circ10_21217626_21219471, suggesting that these axes may play important roles in liver development and its metabolic-immune regulation processes.

### Functional validation of the key competitive endogenous RNA regulatory axis chi-miR-532-3p/circ101504503815046736/CYP8B1

**Figure 5:**
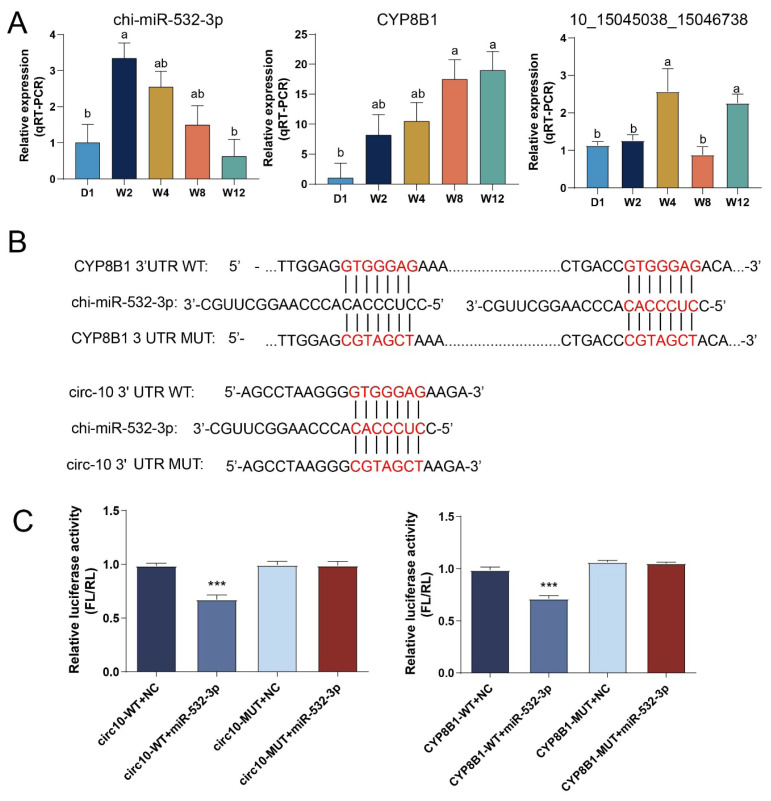
Validation of the competitive endogenous RNA (ceRNA) network. (A) Expression pattern analysis of the chi-miR-532-5p/*CYP8B1*/circ_10_15045038_15046738 axis. (B) Prediction of targeting relationships between chi-miR-532-5p/*CYP8B1* and chi-miR-532-5p/circ_10_ 15045038_15046738. (C) Dual-luciferase assay validation results for the interactions of chi-miR-532-5p with *CYP8B1* and circ_10_15045038_ 15046738. D1, W2, W4, W8, and W12 denote postnatal day 1, and weeks 2, 4, 8, and 12, respectively.

To experimentally validate the regulatory relationships within the key ceRNA axis, the chi-miR-532-3p/circ10_15045038_ 15046736/*CYP8B1* axis was selected for further analysis. qRT-PCR was performed to examine the expression patterns of these three molecules across different developmental stages. The results showed that the expression of chi-miR-532-3p increased initially and then gradually decreased during development, whereas *CYP8B1* expression was continuously up-regulated. The expression of circ10_15045038_15046736 was significantly elevated at W4 and W12 (Figure 5A). Using RNAhybrid, potential complementary binding sites between chi-miR-532-3p and both the *CYP8B1* 3′UTR and circ10_ 15045038_15046736 were predicted (Figure 5B). Dual-luciferase reporter assays (Figure 5C) confirmed that chi-miR-532-3p significantly inhibited the luciferase activity of WT reporters for both *CYP8B1* and circ10_15045038_15046736 (p< 0.01), but had no significant effect on mutant (MUT) constructs, demonstrating that chi-miR-532-3p directly targets both *CYP8B1* and circ10_15045038_15046736.

## DISCUSSION

circRNA, as a conserved non-coding RNA widely present in eukaryotes, has been shown to play important regulatory roles in various biological processes [25,26]. Although they have been confirmed to have significant roles in other species and tissues, the expression patterns of circRNA in the liver of young ruminants with unique digestive and metabolic systems, as well as their regulatory networks, remain completely unexplored. Therefore, this study systematically depicts the circRNA expression characteristics in the liver of Laiwu black goats at five key developmental stages and, through integrating bioinformatics analysis and experimental validation, clarifies their potential regulatory roles, thus providing new insights for understanding the post-transcriptional regulatory mechanisms during the maturation process of ruminant livers.

This study shows that the coordinated evolution of liver function during early development (D1 to W8) may prevent its transcriptome from being clearly distinguished in PCA. In contrast, samples at W12 can be well differentiated from other time points, which may be related to the gradual maturation of liver physiological functions after complete weaning and transition to solid feed. This result is consistent with the characteristic transition of liver transcriptomes in giant pandas from functional specificity during the nursing period to adulthood [27]. In addition, a total of 178 DECs were identified during the early developmental stages after goat birth, with three distinct expression patterns observed. Many circRNA molecules are directly derived from the transcripts of their host genes, and the biological functions of these circRNAs may be closely related to the core functions of their host genes [28,29]. Functional enrichment analysis indicated that the source genes of the DECs from different clusters were significantly enriched in pathways related to immune response, ion homeostasis, lipid metabolism, and cell signaling, suggesting that circRNAs have diverse regulatory functions in liver development. We found that the tryptophan metabolism pathway was significantly enriched in cluster 9. Tryptophan metabolism can participate in energy homeostasis and anti-inflammatory responses through the kynurenine pathway [30]. During the transition from milk to solid feed in lambs, the immune system is not yet mature, and stresses such as weaning can easily induce an inflammatory state [31]. Enhanced tryptophan metabolism may help maintain liver immune homeostasis, and the sustained high expression of circRNAs in cluster 9 suggests that they may alleviate weaning stress in lambs by regulating key genes in the tryptophan metabolism pathway. The DECs in cluster 0 exhibited a trend of continuous downregulation, forming a developmentally related inhibitory regulatory module [32]. This type of circRNA is highly expressed during lactation and may maintain a lactose-dominant nutritional metabolism pattern by suppressing lipid metabolism pathways such as AMPK and insulin signaling pathway [33]. As development matures, the expression of circRNAs in this cluster gradually decreases, possibly facilitating the upregulation of genes involved in fatty acid β-oxidation, gluconeogenesis, and butyrate metabolism by relieving their signaling inhibition on pathways like AMPK pathway [34,35], ultimately guiding the transition of liver metabolism from glucose-dominated to fatty acid-dominated energy utilization.

Recent studies suggest that the biological functions of circRNAs may not only stem from their synergistic interactions with the core functions of host genes but also from their independent regulatory roles mediated as miRNA molecular sponges (ceRNAs) [36]. Based on the ceRNA hypothesis, this study further constructed a ceRNA regulatory network related to liver development, immunity, and metabolism, which includes 20 DECs, 15 DEMs, and 21 DEGs. Among them, the ceRNA axes chi-miR-532-3p/circ10_15045038_15046736/*CYP8B1*, chi-miR-296-3p/*KLF2*/circ10_15045038_15046736, and miR-542-5p/*ACACB*/circ10_21217626_21219471 were identified as potential core regulatory pathways. Among them, chi-miR-532-3p, chi-miR-296-3p, and chi-miR-542-5p show a significant negative correlation with BW and LW, while circ 10_15045038_15046736; 10_21217626_21219471, *CYP8B1*, *KLF2*, and *ACACB* show a significant positive correlation with BW and LW. Notably, overexpression of miR-542-5p in the liver of diabetic model mice significantly reduced blood glucose levels, insulin levels, and lipid accumulation [37,38]. Its target gene *ACACB* is a key regulatory enzyme in lipid metabolism, controlling the balance of fatty acid synthesis and oxidation, and plays a central role in the conversion of glucose and lipid metabolism [39,40]. This study speculates that the miR-542-5p/*ACACB*/circ102121762621219471 axis may play an important role in the early postnatal liver development of goats by regulating glucose and lipid metabolism processes. Additionally, *CYP8B1*, as a key enzyme in bile acid synthesis, holds a core position in maintaining hepatic glucose and lipid metabolism homeostasis and functional maturation [41,42]. In this study, the expression level of *CYP8B1* continuously increased during the postnatal liver maturation process and was significantly enriched in the primary bile acid biosynthesis pathway, indicating that the liver bile acid synthesis pathway may be significantly activated, marking the gradual maturation of hepatocyte metabolic function. Its expression dynamics are consistent with the body’s demand for lipid digestion and absorption after weaning, suggesting that *CYP8B1* may influence lipid metabolism by regulating bile acid composition, thereby promoting metabolic transition in the liver during early developmental changes related to diet. Meanwhile, circRNA 10_15045038_15046738 is derived from the Vasohibin 1 (*VASH1*) gene. Previous studies have indicated that low expression of *VASH1* can lead to decreased insulin levels and increased blood glucose in mice, further supporting the potential role of this circRNA in regulating glucose and lipid metabolism [43]. In this study, its expression at W4 and W12 was significantly higher than at other stages, indicating that it may be involved in the adaptive regulation of liver glucose and lipid metabolism during the early developmental process of goats. It is worth noting that chi-miR-532-3p has been confirmed to participate in the post-transcriptional regulation of gluconeogenesis in pigs [44]. The above results indicate that chi-miR-532-3p/circ1015045038_15046736/*CYP8B1* may constitute a key ceRNA regulatory axis: circ1015045038_ 15046736 may release the inhibitory effect of chi-miR-532-3p on *CYP8B1* by adsorbing chi-miR-532-3p, thereby promoting the expression of *CYP8B1* at the post-transcriptional level, ultimately coordinating the regulation of bile acid synthesis and glucose-lipid metabolism balance in the liver. This study also further confirmed the targeted regulatory relationship among chi-miR-532-3p/circ10_15045038_15046736/*CYP8B1* through dual-luciferase reporter gene experiments.

This study still has certain limitations, as it did not measure physiological data such as blood biochemical indicators and integrate them with this study for analysis. In addition, to fully validate these key ceRNA axes and clarify their precise biological mechanisms and physiological significance, further functional experiments are still needed.

## CONCLUSION

This study systematically analyzes the dynamic expression profile of circRNA during the developmental process of young goat liver. Through functional enrichment analysis and the construction of a ceRNA regulatory network, it reveals that circRNA may participate in regulating key biological processes such as lipid metabolism, immune response, and cell signal transduction through its source genes. In addition, key ceRNA networks like chi-miR-532-3p/circ10_15045038_15046736/*CYP8B1* and miR-542-5p/*ACACB*/circ10_21217626_ 21219471 may influence liver metabolism and immune homeostasis at the post-transcriptional level. These results not only enhance the understanding of post-transcriptional regulatory mechanisms in early liver development of ruminants but also provide a new theoretical basis and research perspective for further exploring the role of circRNA in liver physiological and pathological processes.

## Data Availability

The dataset(s) supporting the conclusions of this article is(are) available in the NCBI database. Reference number: GSE194190 and PRJNA770352.

## SUPPLEMENTARY MATERIAL

**Supplement 1**. Determination of body weight and liver weights in goats.

**Supplement 2**. Primers for validation and qPCR.

**Supplement 3**. Summary of reads mapped to the goat genome.

**Supplement 4**. Identification of differentially expressed circular RNAs during early development in goat live.

**Supplement 5**. Correlation analysis of miRNA, mRNA, and circRNA within the ceRNA regulatory network.

**Supplement 6**. KEGG enrichment analysis of DEGs in the ceRNA network.

## References

[1] Han HS Kang G Kim JS Choi BH Koo SH Regulation of glucose metabolism from a liver-centric perspective Exp Mol Med 2016 48 e218 10.1038/emm.2015.122 26964834 PMC4892876

[2] Kubes P Jenne C Immune responses in the liver Annu Rev Immunol 2018 36 247 77 10.1146/annurev-immunol-051116-052415 29328785

[3] Trefts E Gannon M Wasserman DH The liver Curr Biol 2017 27 R1147 51 10.1016/j.cub.2017.09.019 29112863 PMC5897118

[4] Spear BT Jin L Ramasamy S Dobierzewska A Transcriptional control in the mammalian liver: liver development, perinatal repression, and zonal gene regulation Cell Mol Life Sci 2006 63 2922 38 10.1007/s00018-006-6258-5 17041810 PMC11136251

[5] Kristensen LS Andersen MS Stagsted LVW Ebbesen KK Hansen TB Kjems J The biogenesis, biology and characterization of circular RNAs Nat Rev Genet 2019 20 675 91 10.1038/s41576-019-0158-7 31395983

[6] Misir S Wu N Yang BB Specific expression and functions of circular RNAs Cell Death Differ 2022 29 481 91 10.1038/s41418-022-00948-7 35169296 PMC8901656

[7] Tay Y Rinn J Pandolfi PP The multilayered complexity of ceRNA crosstalk and competition Nature 2014 505 344 52 10.1038/nature12986 24429633 PMC4113481

[8] Liu J Guo C Fu J Identification and functional analysis of circRNAs during goat follicular development Int J Mol Sci 2024 25 7548 10.3390/ijms25147548 39062792 PMC11277404

[9] Zhang X Zhan S Yang S Dynamic expression profiles of circular RNAs during brown to white adipose tissue transformation in goats (Capra hircus) Animals 2021 11 1351 10.3390/ani11051351 34068539 PMC8150810

[10] Zhou Z Li K Liu J Expression profile analysis to identify circular RNA expression signatures in muscle development of Wu’an goat longissimus dorsi tissues Front Vet Sci 2022 9 833946 10.3389/fvets.2022.833946 35518637 PMC9062782

[11] Chen S Zhou Y Chen Y Gu J fastp: an ultra-fast all-in-one FASTQ preprocessor Bioinformatics 2018 34 i884 90 10.1093/bioinformatics/bty560 30423086 PMC6129281

[12] Thakur V RNA-seq data analysis for differential gene expression using HISAT2–StringTie–Ballgown pipeline Azad RK Transcriptome data analysis Humana 2024 101 13 10.1007/978-1-0716-3886-6_5 39068358

[13] Pertea M Pertea GM Antonescu CM Chang TC Mendell JT Salzberg SL StringTie enables improved reconstruction of a transcriptome from RNA-seq reads Nat Biotechnol 2015 33 290 5 10.1038/nbt.3122 25690850 PMC4643835

[14] Gao Y Wang J Zhao F CIRI: an efficient and unbiased algorithm for de novo circular RNA identification Genome Biol 2015 16 4 10.1186/s13059-014-0571-3 PMC4316645 25583365

[15] Liang J Li C Zhang Y The integration of WGCNA and ceRNA analysis provides insights into bovine intramuscular fat deposition BMC Genom 2025 26 865 10.1186/s12864-025-12097-5 PMC12482651 41023666

[16] Love MI Huber W Anders S Moderated estimation of fold change and dispersion for RNA-seq data with DESeq2 Genome Biol 2014 15 550 10.1186/s13059-014-0550-8 25516281 PMC4302049

[17] Ernst J Bar-Joseph Z STEM: a tool for the analysis of short time series gene expression data BMC Bioinform 2006 7 191 10.1186/1471-2105-7-191 PMC1456994 16597342

[18] Xu S Hu E Cai Y Using clusterProfiler to characterize multiomics data Nat Protoc 2024 19 3292 320 10.1038/s41596-024-01020-z 39019974

[19] Bu D Luo H Huo P KOBAS-i: intelligent prioritization and exploratory visualization of biological functions for gene enrichment analysis Nucleic Acids Res 2021 49 W317 25 10.1093/nar/gkab447 34086934 PMC8265193

[20] Zhao X Xuan R Wang A High-throughput sequencing reveals transcriptome signature of early liver development in goat kids Genes 2022 13 833 10.3390/genes13050833 35627218 PMC9141777

[21] Zhao X Ji Z Xuan R Characterization of the microRNA expression profiles in the goat kid liver Front Genet 2022 12 794157 10.3389/fgene.2021.794157 35082837 PMC8784682

[22] Enright AJ John B Gaul U Tuschl T Sander C Marks DS MicroRNA targets in Drosophila Genome Biol 2003 5 R1 10.1186/gb-2003-5-1-r1 14709173 PMC395733

[23] Krüger J Rehmsmeier M RNAhybrid: microRNA target prediction easy, fast and flexible Nucleic Acids Res 2006 34 W451 4 10.1093/nar/gkl243 16845047 PMC1538877

[24] Ahlawat S Vasu M Choudhary V Comprehensive evaluation and validation of optimal reference genes for normalization of qPCR data in different caprine tissues Mol Biol Rep 2024 51 268 10.1007/s11033-024-09268-0 38302649

[25] Chen W Xu J Wu Y The potential role and mechanism of circRNA/miRNA axis in cholesterol synthesis Int J Biol Sci 2023 19 2879 96 10.7150/ijbs.84994 37324939 PMC10266072

[26] Zhang P Dai M CircRNA: a rising star in plant biology J Genet Genomics 2022 49 1081 92 10.1016/j.jgg.2022.05.004 35644325

[27] Ma J Shen F Chen L Gene expression profiles during postnatal development of the liver and pancreas in giant pandas Aging 2020 12 15705 29 10.18632/aging.103783 32805731 PMC7467380

[28] Cao M Yuan GH Cao SM Direct circMAN1A2(2,3,4,5)-CENPB mRNA interaction regulates cell proliferation and cancer progression Nat Commun 2025 16 8609 10.1038/s41467-025-63686-7 41022764 PMC12480695

[29] You X Vlatkovic I Babic A Neural circular RNAs are derived from synaptic genes and regulated by development and plasticity Nat Neurosci 2015 18 603 10 10.1038/nn.3975 25714049 PMC4376664

[30] Cervenka I Agudelo LZ Ruas JL Kynurenines: tryptophan’s metabolites in exercise, inflammation, and mental health Science 2017 357 eaaf9794 10.1126/science.aaf9794 28751584

[31] Li Y Han L Liu J Kang L Zhao L Cui K Yeast peptides improve the intestinal barrier function and alleviate weaning stress by changing the intestinal microflora structure of weaned lambs Microorganisms 2023 11 2472 10.3390/microorganisms11102472 37894129 PMC10608930

[32] Wang B Cui L Song Q Excessive dietary L-tryptophan regulated amino acids metabolism and serotonin signaling in the colon of weaning piglets with acetate-induced gut inflammation Amino Acids 2023 55 403 12 10.1007/s00726-023-03239-8 36648538

[33] Yan Y Mukherjee S Harikumar KG Structure of an AMPK complex in an inactive, ATP-bound state Science 2021 373 413 9 10.1126/science.abe7565 34437114 PMC8428800

[34] Zhou X Yang H Yan Q Evidence for liver energy metabolism programming in offspring subjected to intrauterine undernutrition during midgestation Nutr Metab 2019 16 20 10.1186/s12986-019-0346-7 PMC6423887 30923555

[35] Brown LD Kohn JR Rozance PJ Hay WW Jr Wesolowski SR Exogenous amino acids suppress glucose oxidation and potentiate hepatic glucose production in late gestation fetal sheep Am J Physiol Regul Integr Comp Physiol 2017 312 R654 63 10.1152/ajpregu.00502.2016 28179229 PMC5451575

[36] Zhang C Yu Z Yang S ZNF460-mediated circRPPH1 promotes TNBC progression through ITGA5-induced FAK/PI3K/AKT activation in a ceRNA manner Mol Cancer 2024 23 33 10.1186/s12943-024-01944-w 38355583 PMC10865535

[37] Tian F Ying HM Wang YY Cheng BN Chen J MiR-542–5p inhibits hyperglycemia and hyperlipoidemia by targeting FOXO1 in the liver Yonsei Med J 2020 61 780 8 10.3349/ymj.2020.61.9.780 32882762 PMC7471073

[38] Guo N Nulahou A Bu Q MiR-542-5p regulates the progression of diabetic retinopathy by targeting CARM1 Acta Biochim Pol 2020 67 373 8 10.18388/abp.2020_5228 32870623

[39] Deja S Fletcher JA Kim CW Hepatic malonyl-CoA synthesis restrains gluconeogenesis by suppressing fat oxidation, pyruvate carboxylation, and amino acid availability Cell Metab 2024 36 1088 104.E12 10.1016/j.cmet.2024.02.004 38447582 PMC11081827

[40] Lee AK Kyriakou T Weston AJ O’Dell SD Functional single-nucleotide polymorphism in acetyl-CoA carboxylase ACACB gene promoter DNA Cell Biol 2010 29 703 12 10.1089/dna.2010.1078 20799892

[41] Liu J Liu Y Huang C Quercetin-driven Akkermansia muciniphila alleviates obesity by modulating bile acid metabolism via an ILA/m6A/CYP8B1 signaling Adv Sci 2025 12 2412865 10.1002/advs.202412865 PMC11948036 39888270

[42] Kaur A Patankar JV de Haan W Loss of Cyp8b1 improves glucose homeostasis by increasing GLP-1 Diabetes 2015 64 1168 79 10.2337/db14-0716 25338812

[43] Tang XW Qin QX miR-335-5p induces insulin resistance and pancreatic islet β-cell secretion in gestational diabetes mellitus mice through VASH1-mediated TGF-β signaling pathway J Cell Physiol 2019 234 6654 66 10.1002/jcp.27406 30341900

[44] Jia Y Cong R Li R Maternal low-protein diet induces gender-dependent changes in epigenetic regulation of the glucose-6-phosphatase gene in newborn piglet liver J Nutr 2012 142 1659 65 10.3945/jn.112.160341 22833655

