# Supplementary material for: Comprehensive analysis of circular RNA expression dynamics and competitive endogenous RNA network mechanisms during postnatal liver development in juvenile goats
Source: Anim Biosci. 2025 Nov 25;39(4):250689. doi: 10.5713/ab.250689 (PMC13064993; doi:10.5713/ab.250689)
Supplement: Supplementary file 1 [file ab-250689-Supplementary-1.pdf]

Supplement 1. Determination of body weight and liver weights in goats.

sample	Body weigh	Liver weight(g)
D1_1	4.2	39.69
D1_2	3.65	122.67
D1_3	2.1	71.53
D1_4	3.05	102.94
D1_5	2.9	64.34
W2_1	2.85	101.6
W2_2	3.85	168.13
W2_3	5.2	132.56
W2_4	4.65	133.82
W2_5	3.85	95.65
W4_1	4.15	105.18
W4_2	4.4	104.88
W4_3	4.25	138.77
W4_4	4.45	132.2
W4_5	4.25	108.1
W8_1	8.55	141.49
W8_2	7.15	161.91
W8_3	7.25	157.99
W8_4	4	166.43
W8_5	4.7	113.47
W12_1	11.25	195.51
W12_2	10.45	211.24
W12_3	9.7	165.87
W12_4	10.65	238.81
W12_5	11.35	239.74
