# Supplementary material for: Comprehensive analysis of circular RNA expression dynamics and competitive endogenous RNA network mechanisms during postnatal liver development in juvenile goats
Source: Anim Biosci. 2025 Nov 25;39(4):250689. doi: 10.5713/ab.250689 (PMC13064993; doi:10.5713/ab.250689)
Supplement: Supplementary file 2 [file ab-250689-Supplementary-2.pdf]

Supplement 2. Primers for validation and qPCR

Primer name	Nucleotide sequence 5'_3’
CIRCRNA_5_102270759_102311752-F	CAACAGGGGAACAAGGCACTC
CIRCRNA_5_102270759_102311752-R	TCTCTGGTCGTGTGATTGAAGG
CIRCRNA_24_47713523_47745509-F	CTGGTGGAGTGAATGGCAAGA
CIRCRNA_24_47713523_47745509-R	AGTGAAGATGGAGAAACAAGCGAT
CIRCRNA_10_15045038_15046738-F	CAAAGAACTGTGTCCCTGTGTGAT
CIRCRNA_10_15045038_15046738-R	CATGACTCTGCCTTCCAACCC
CIRCRNA_10_21217626_21219471-F	CAAGGGTGGTGGTGAGGATTT
CIRCRNA_10_21217626_21219471-R	ACGCCATACACAAGACTTAGCC
β-Actin-F	CATGTACGTTGCTATCCAGGC
β-Actin-R	CTCCTTAATGTCACGCACGAT
CYP8B1-F	CGCAAGTATGACCGCCTGTTCC
CYP8B1-R	GCCGTACTTCTCCAGGTTGTGTTC
chi-miR-532-3p-F	CTCCCACACCCAAGGCTTG
