# Supplementary material for: Comprehensive analysis of circular RNA expression dynamics and competitive endogenous RNA network mechanisms during postnatal liver development in juvenile goats
Source: Anim Biosci. 2025 Nov 25;39(4):250689. doi: 10.5713/ab.250689 (PMC13064993; doi:10.5713/ab.250689)
Supplement: Supplementary file 3 [file ab-250689-Supplementary-3.pdf]

Supplement 3. Summary of reads mapped to the goat genome

Sample	RawReads	CleanReads	Q20(%)	Q30(%)	GCcontent (%)	Total mapped
D1_1	124706552	122652950	97.73	93.26	50.11	119526217(97.45%)
D1_2	124711436	121783280	97.57	93.06	51.29	118365352(97.19%)
D1_3	121605834	119676092	97.65	93.25	50.11	116299281(97.18%)
D1_4	125007672	120264076	97.55	93	50.57	117157837(97.42%)
D1_5	123313310	120479764	97.73	93.39	51.17	117342955(97.4%)
W2_1	123078860	120859436	97.72	93.4	51.45	117162548(96.94%)
W2_2	125724416	123705754	97.73	93.39	50.39	120370171(97.3%)
W2_3	120567842	118304356	97.62	93.13	50.06	115202551(97.38%)
W2_4	162766876	136724132	97.7	93.25	50.87	132755116(97.1%)
W2_5	124667212	122445796	97.6	93.12	50.47	119027235(97.21%)
W4_1	122695698	120410174	97.51	92.98	50.46	116902441(97.09%)
W4_2	119138166	117388094	97.63	93.16	50.82	114165989(97.26%)
W4_3	122019306	120185204	97.58	93.09	50.54	116771445(97.16%)
W4_4	124607136	123101152	97.81	93.54	51.05	119636922(97.19%)
W4_5	125188728	123580984	97.76	93.39	50.59	120356723(97.39%)
W8_1	124239242	121955756	97.86	93.7	50.97	118574248(97.23%)
W8_2	120650752	119109088	97.83	93.55	50.84	116160798(97.52%)
W8_3	117802766	115449396	97.76	93.38	50.21	112576636(97.51%)
W8_4	121776430	119620226	97.74	93.34	50.8	116392152(97.3%)
W8_5	125408616	120674068	97.69	93.25	50.94	117566378(97.42%)
W12_1	120588066	119033274	97.81	93.55	51.73	115840121(97.32%)
W12_2	122109328	119799184	97.43	92.52	51.05	116198233(96.99%)
W12_3	122277196	119656710	97.78	93.46	50.12	116673515(97.51%)
W12_4	117731312	114642794	97.77	93.44	51	111662013(97.4%)
W12_5	124226524	122363858	97.75	93.33	50.09	119161730(97.38%)

Multiple mapped	Unique mapped
11359329(9.26%)	108166888(88.19%)
11693696(9.6%)	106671656(87.59%)
11002117(9.19%)	105297164(87.99%)
12697214(10.56%)	104460623(86.86%)
12589812(10.45%)	104753143(86.95%)
12277063(10.16%)	104885485(86.78%)
12269044(9.92%)	108101127(87.39%)
12025689(10.17%)	103176862(87.21%)
15714255(11.49%)	117040861(85.6%)
12710898(10.38%)	106316337(86.83%)
11231241(9.33%)	105671200(87.76%)
10431646(8.89%)	103734343(88.37%)
12150598(10.11%)	104620847(87.05%)
12345381(10.03%)	107291541(87.16%)
12473458(10.09%)	107883265(87.3%)
13868028(11.37%)	104706220(85.86%)
13173902(11.06%)	102986896(86.46%)
12046959(10.43%)	100529677(87.08%)
13430024(11.23%)	102962128(86.07%)
12965052(10.74%)	104601326(86.68%)
13042473(10.96%)	102797648(86.36%)
11636839(9.71%)	104561394(87.28%)
10534177(8.8%)	106139338(88.7%)
11230955(9.8%)	100431058(87.6%)
10878213(8.89%)	108283517(88.49%)
