# Supplementary material for: Comprehensive analysis of circular RNA expression dynamics and competitive endogenous RNA network mechanisms during postnatal liver development in juvenile goats
Source: Anim Biosci. 2025 Nov 25;39(4):250689. doi: 10.5713/ab.250689 (PMC13064993; doi:10.5713/ab.250689)
Supplement: Supplementary file 4 [file ab-250689-Supplementary-4.pdf]

<u>Supplement 4.</u> Identification of differentially expressed circular RNAs during early development in goat live

	circRNA id	log2FoldChange	pvalue	padj
	10_89824027_8984090	-4.26978918	0.00064963	0.040892124
	11_78230041_7824484	-1.043727248	0.00056401	0.036728768
	12_50459455_5048068	-1.401740115	0.00033773	0.027914606
	18_40246240_4024724	-21.03366023	6.22E-06	0.002229537
	19_25059774_2507009	-1.496108461	0.00040155	0.030818927
	19_25059778_2507009	-4.893246545	0.0002069	0.020756502
	1_67221974_67226579	-4.706170577	0.00074688	0.04115484
	20_11482602_1148331	-2.835917761	0.00029877	0.027475508
	20_6820353_6824596	-2.439982161	0.00074182	0.04115484
	21_41984105_4198909	-3.00704266	0.00069494	0.04115484
	21_58403056_5840883	-5.862570366	4.24E-06	0.00182074
	23_17926944_1793860	-2.085346369	6.73E-05	0.0096445
	24_61335363_6134400	-2.350430834	0.00010589	0.012642559
	4_27166115_27168870	-1.929895394	0.00077292	0.041525088
	5_112048568_1120670	-9.53011226	0.00010541	0.012642559
	6_68272249_68285449	-2.094872869	2.39E-05	0.006070464
	7_92721333_92726238	-5.020167015	5.36E-05	0.008231848
	8_94379232_94394086	-2.813901294	2.94E-07	0.000210529
	8_94379232_94407339	-1.386274215	0.00073715	0.04115484
	9_48115022_48125137	-1.597641561	0.00031963	0.027475508
	LWLT01000027.1_113	-1.368277349	0.00095439	0.048832958
W2 vs. D1	LWLT01000027.1_367	-3.615404342	0.00053212	0.036728768
	10_35997432_3599795	2.298888928	0.00013526	0.014533875
	13_43061823_4311019	7.140938262	0.00099242	0.049419777
	16_74953105_7495401	1.098495078	0.00021249	0.020756502
	19_32516428_3252049	1.767438275	4.09E-05	0.007551874
	19_42531815_4258968	4.278441321	0.00085405	0.04476471
	1_117855695_1178982	4.724348376	0.00101185	0.049419777
	21_41938603_4196389	1.326439287	2.21E-05	0.006070464
	22_35231465_3523771	3.800628453	0.00039442	0.030818927
	22_54855072_5487838	1.413592723	0.00012573	0.0142212
	23_23531594_2358109	21.8165316	4.67E-14	5.02E-11
	24_45998215_4599954	4.936086065	7.61E-05	0.010224183
	24_58188981_5820321	1.339429273	2.82E-05	0.006070464
	26_10158184_1023752	4.812370377	0.00053203	0.036728768
	26_35338092_3534091	5.752902604	3.09E-31	6.65E-28
	26_7728428_7737431	2.369173277	1.20E-06	0.000643566
	28_20841606_2085840	5.282042037	5.18E-05	0.008231848
	29_51170118_5117358	2.357309453	2.72E-05	0.006070464
	2_29788651_29798577	4.953881402	4.22E-05	0.007551874
	3_34884799_34895626	4.629003979	0.00031566	0.027475508
	4_1238642_1253045	4.669513307	0.00055898	0.036728768
	7_101322477_1013750	2.849213444	0.000666	0.040892124
	10_15045038_1504673	3.01312317	7.92E-05	0.008082696
	10_35997432_3599795	2.042742537	0.00070983	0.034375514
	10_55045668_5506915	2.406132128	0.0010844	0.046572092
	10_97941407_9794182	4.181165301	0.00017021	0.014620213
	11_283882_295588	1.414020004	5.58E-05	0.006107543
	12_7369087_7396799	1.335096873	0.00053855	0.033995256
	13_43061823_4311019	8.174574449	0.00016061	0.014562141
	14_21977748_2200373	2.937236667	0.00012488	0.011988004
	14_45082123_4516767	1.427492009	0.00066066	0.034375514
	16_70332468_7034440	1.353721513	0.00019694	0.015304731
	17_27735333_2773806	1.377149705	0.00086169	0.040179374

	17_7243729_7243998	5.213669715	2.39E-05	0.00300447
	19_42531815_4258968	4.33163726	0.00066692	0.034375514
	22_35231465_3523771	3.973586511	0.00018289	0.014923779
	22_54855072_5487838	1.891826145	2.00E-07	5.45E-05
	23_23531594_2358109	22.13869882	1.97E-14	1.07E-11
	26_35338092_3534091	6.560012645	2.14E-40	3.50E-37
	28_40705332_4072480	1.449196794	0.00091769	0.041601878
	2_29788651_29798577	4.266129831	0.0004934	0.033550961
W4 vs. D1	3_114116822_1141227	4.053565035	0.00063451	0.034375514
	3_34884799_34895626	6.884097848	3.39E-08	1.11E-05
	3_34889318_34895626	4.080348351	3.43E-06	0.000621332
	5_15084500_15085511	4.21708467	0.00098416	0.043409383
	11_62033384_6203445	-1.347861316	5.61E-05	0.006107543
	12_50459455_5048068	-1.712818034	1.42E-05	0.002108344
	18_40246240_4024724	-21.39123955	4.32E-06	0.000704818
	18_55779595_5580175	-23.30627845	5.47E-07	0.000127604
	1_118567791_1185714	-1.295931853	0.00054159	0.033995256
	1_118567791_1185740	-2.82464721	0.00027084	0.020091364
	23_28430415_2843308	-2.280007348	0.00071616	0.034375514
	25_37565394_3761808	-20.42011741	1.39E-15	1.14E-12
	2_107548050_1075752	-6.396073936	0.00036538	0.025925872
	2_14462575_14463591	-4.447389252	0.0006857	0.034375514
	8_94379232_94394086	-2.457395102	1.61E-06	0.000329109
	8_94379232_94407339	-2.730599376	3.47E-10	1.41E-07
	8_94505434_94527149	-3.215058614	0.00069996	0.034375514
	9_48115022_48125137	-1.357776902	0.00117455	0.049150265
	LWLT01000027.1_367	-3.306637509	0.00061102	0.034375514
	LWLT01000044.1_268	-3.110094304	1.74E-05	0.00237057
	15_57753770_5775512	-1.983566629	0.00034378	0.048631925
	17_2211680_2212292	-1.743459625	8.05E-05	0.021138861
	18_55779595_5580175	-25.91910779	2.53E-08	2.33E-05
	19_39526039_3952623	-4.127842521	0.00016869	0.02820186
	1_134405843_1344085	-1.5883653	6.23E-05	0.019085457
	25_37565394_3761808	-26.2967153	5.76E-25	1.06E-21
	7_934615_939167	-1.178297166	2.54E-05	0.009330289
W4 vs. W2	10_89824027_8984090	4.720922534	0.00014915	0.027427724
	13_29700413_2973183	1.347836006	0.00011283	0.025936304
	14_55667646_5568221	4.702790175	0.00013971	0.027427724
	21_41984105_4198909	3.2837322	0.00020322	0.031143639
	2_118629711_1186497	2.30366641	7.72E-07	0.000473326
	5_112048568_1120670	11.22444504	4.93E-06	0.002266307
	7_92721333_92726238	4.471330278	0.0003709	0.048720762
	11_62033384_6203445	-1.034236565	0.00184705	0.045567816
	12_50459455_5048068	-1.533935577	8.47E-05	0.005858892
	12_70964537_7096587	-2.101652254	0.00023245	0.010457064
	12_74495943_7455364	-1.809988881	0.00108716	0.033526159
	14_45049116_4509150	-1.308943591	0.00022384	0.010431084
	14_54949652_5495206	-1.592921429	0.00017026	0.009068128
	15_50440234_5044590	-1.890568454	0.00016736	0.009068128
	16_4481845_4562530	-26.44582999	6.00E-11	3.22E-08
	16_72097313_7210078	-4.560615742	0.00215133	0.049935684
	19_10321979_1032891	-2.193257956	0.00139748	0.037935601
	19_25059774_2507009	-2.285262635	6.70E-07	0.000200839
	19_25059774_2509929	-4.87609963	5.25E-05	0.004586631
	1_118567791_1185714	-1.20702705	0.00126576	0.036865315
	1_135223138_1352283	-1.64645066	0.00058136	0.021769688

	1_65060844_65065945	-2.156788776	0.00191132	0.046098943
	21_19906737_1991000	-1.951614504	0.00018148	0.009157689
	21_58403056_5840883	-6.016680682	2.35E-06	0.000493721
	24_56452379_5645337	-3.76573485	0.00091904	0.029714141
	25_7777603_7777878	-1.594852326	0.00117895	0.034820664
	26_19060061_1906314	-1.911793994	0.00147744	0.039217703
	28_36619236_3662492	-1.837382124	4.56E-05	0.004186628
	29_35660301_3566137	-2.962413384	0.00207482	0.048886398
	3_28477370_28481761	-3.5615412	0.00018538	0.009157689
	3_72167775_72194318	-2.269254559	5.56E-06	0.000896075
	3_74789455_74824821	-2.662695574	0.00110969	0.033725021
	3_78123006_78130924	-1.066438707	0.00018778	0.009157689
	3_98013885_98035705	-1.477373323	0.00050357	0.020307505
	3_98028970_98035705	-2.723512106	0.00064513	0.023324744
	5_112048568_1120670	-8.530070165	0.00051879	0.020526372
	6_22759655_22769390	-1.404486457	0.0015404	0.040293578
	6_68272249_68299586	-1.238053977	1.19E-05	0.001663958
	8_62162323_62163643	-3.45873961	0.00114304	0.034242137
	8_94379232_94394086	-1.960112143	6.84E-05	0.005119025
	8_94379232_94407339	-1.613810744	8.94E-05	0.005858892
	LWLT01000027.1_113	-1.973640765	5.39E-06	0.000896075
	LWLT01000027.1_367	-3.413154755	0.00055128	0.021408143
	LWLT01001839.1_152	-6.543781097	6.12E-05	0.004755843
	10_1010403_1013814	2.170553731	0.00010299	0.006351788
	10_15045038_1504673	3.109837784	4.59E-05	0.004186628
	10_21217626_2121947	5.409015576	2.38E-05	0.002774397
	10_35997432_3599795	2.5746088	1.35E-05	0.001773626
	10_49128465_4916891	2.767097479	0.00082272	0.028282825
	10_49139664_4914694	3.90554165	0.00034302	0.014386053
	10_55045668_5506915	2.920466632	6.10E-05	0.004755843
W8 vs. D1	10_97941407_9794182	5.157915811	2.70E-06	0.000514303
	11_283882_295588	1.46673754	2.96E-05	0.003264687
	12_58563987_5857037	4.003624357	0.00031756	0.013590205
	13_43061823_4311019	7.382519138	0.00065972	0.023447846
	14_21977748_2200373	2.795959199	0.00027832	0.012159111
	14_71447243_7146580	1.759880916	0.00157562	0.040293578
	15_17934272_1794001	1.779030605	0.00095507	0.030234673
	16_5233603_5234968	2.73119908	8.16E-05	0.005858892
	16_70332468_7034440	1.553215376	1.83E-05	0.002256152
	17_13083954_1310315	1.012027234	0.00136441	0.037935601
	17_7243729_7243998	5.097866094	3.82E-05	0.004004194
	19_32516428_3252049	1.356075488	0.0019152	0.046098943
	19_42531815_4258968	4.370052355	0.00060358	0.02220548
	1_117843726_1178982	4.374045281	0.00014664	0.008541723
	1_117855695_1178982	4.779467774	0.0008467	0.028637679
	22_35231465_3523771	5.135097953	8.74E-07	0.000229049
	22_54855072_5485889	4.310746793	0.00023437	0.010457064
	22_54855072_5487838	2.367478876	6.15E-11	3.22E-08
	22_54855072_5488951	4.334914499	0.00129667	0.03724828
	22_54855072_5491339	1.823465436	0.00021505	0.01024888
	23_23531594_2358109	21.45279196	1.22E-13	1.28E-10
	24_58188981_5820321	1.000393607	0.00193453	0.046098943
	26_10455772_1047954	1.852740654	0.00160981	0.040672042
	26_35338092_3534091	6.612971053	5.12E-41	1.07E-37
	26_7728428_7737431	2.642444933	3.92E-08	1.65E-05
	28_20841606_2085840	4.267696327	0.00131479	0.037258199

	28_40705332_4072480	1.448818053	0.00096601	0.030234673
	29_51170118_5117358	2.185097261	0.00010116	0.006351788
	2_116645728_1166488	1.770767788	8.87E-05	0.005858892
	2_29788651_29798577	4.848227047	6.03E-05	0.004755843
	2_50314931_50324935	1.510297508	0.00137721	0.037935601
	2_9397149_9398016	3.988458478	0.00141105	0.037935601
	3_114116822_1141227	5.689482651	1.32E-06	0.000307899
	3_34884799_34895626	6.282250168	5.25E-07	0.000183397
	3_34889318_34895626	3.626948492	4.20E-05	0.004186628
	3_34985049_35013916	4.764922606	0.00047505	0.019532968
	4_25407810_25451926	4.932196508	0.00017297	0.009068128
	5_115881622_1158850	1.597635229	0.00057899	0.021769688
	5_15084500_15085511	5.641251371	6.14E-06	0.000919702
	5_90267552_90297427	3.990151815	0.00169585	0.042335597
	7_92969600_92969988	1.59496453	0.00017106	0.009068128
	7_94327801_94328072	1.190971083	0.00012868	0.007709648
	8_99251909_99263537	4.740205213	0.00155793	0.040293578
	8_99443219_99445631	1.584007344	0.00088215	0.029363051
W8 vs. W2	16_4481845_4562530	-26.04880909	1.15E-10	3.08E-07
	18_40246240_4024724	23.06255459	7.19E-07	0.000957942
	21_58790959_5884165	9.041084495	2.30E-06	0.002042386
	7_92721333_92726238	5.445043263	1.12E-05	0.007479066
W8 vs. W4	16_28655771_2866246	-1.889473741	0.00011669	0.03374296
	16_4481845_4562530	-15.55432706	0.00017386	0.037706851
	21_19906737_1991000	-2.062669919	7.69E-05	0.026674256
	5_112048568_1120670	-10.22440294	3.17E-05	0.013755516
	16_4847120_4850019	1.620540165	0.0003007	0.049256545
	18_40246240_4024724	23.4201339	4.83E-07	0.000279104
	18_55779595_5580175	24.84125864	9.34E-08	8.10E-05
	19_39526039_3952623	4.065646153	0.00020793	0.040083951
	25_37565394_3761808	26.59810879	1.67E-25	2.89E-22
	2_14462575_14463591	4.941678712	0.00014958	0.037074274
	3_86771940_86828871	3.408638389	0.00031229	0.049256545
	11_3428426_3458741	-4.123093599	0.00048499	0.02359327
	11_45319520_4532586	-4.106330229	0.00072562	0.030396845
	11_62033384_6203445	-1.484439817	9.97E-06	0.001263245
	12_50459455_5048068	-1.720537326	1.28E-05	0.00140442
	14_45049116_4509150	-1.717400391	3.27E-06	0.000502526
	18_55779595_5580175	-23.34249863	5.26E-07	0.000104877
	19_13892761_1389286	-6.237572077	0.00011162	0.008035897
	19_25059774_2507009	-1.606674628	0.00011567	0.008035897
	19_25059778_2507009	-5.130197676	0.0001002	0.007960639
	19_40184021_4018647	-4.301444916	0.00041462	0.020766877
	19_42521928_4255575	-23.18098229	1.63E-07	5.37E-05
	1_118567791_1185740	-2.611811345	0.00049887	0.02359327
	21_58403056_5840883	-4.945366875	0.00010452	0.008027022
	21_60996150_6102184	-1.592007906	0.00126668	0.042295951
	23_28430415_2843308	-2.360080929	0.00051201	0.02359327
	24_2399338_2411998	-1.602591395	3.79E-05	0.003493849
	26_19065417_1907152	-4.763398182	0.00012666	0.008105953
	28_36619236_3662492	-1.995967533	1.19E-05	0.001372083
	29_9003287_9021328	-1.364111283	0.00106907	0.037320118
	2_113507839_1135091	-2.167914528	0.00096807	0.035403599
	2_3397680_3426072	-5.162322352	0.00152902	0.0470471
	3_28477370_28481761	-3.626607249	0.00013381	0.00828667
	3_41133689_41148202	-4.952607664	2.80E-05	0.002687535

	3_41133689_41155760	-6.16088545	3.69E-08	2.13E-05
	3_41133689_41157200	-3.17978568	5.93E-05	0.005058586
	3_74789455_74824821	-2.496218672	0.0016554	0.048946551
	3_91153335_91154412	-2.249760609	0.00115544	0.039149142
	3_98013885_98035705	-1.309275787	0.0014384	0.045398212
	6_22759655_22769390	-1.452147881	0.0010175	0.036629958
	8_94379232_94394086	-1.867002438	0.00011988	0.008035897
	LWLT01000027.1_113	-2.506446381	4.84E-08	2.23E-05
	LWLT01000027.1_113	-5.261559096	0.00134666	0.04369989
	LWLT01001839.1_152	-6.626621879	4.93E-05	0.004372061
	10_15045038_1504673	2.740225695	0.00036074	0.018469752
	10_21217626_2121947	4.908709285	0.00013838	0.00828667
	10_49139664_4914694	4.119374944	0.00014483	0.008342197
	10_55045668_5506915	2.45470104	0.00084034	0.032268947
	10_97941407_9794182	4.85618903	1.04E-05	0.001263245
	11_102883877_102885	1.216236036	8.77E-05	0.00721466
W12 vs. D1	11_283882_295588	1.775687242	2.57E-07	6.59E-05
	11_59735509_5974746	1.670546721	0.00165705	0.048946551
	12_7369087_7396799	1.379522754	0.0003386	0.017730217
	13_43061823_4311019	9.758359239	6.54E-06	0.000886744
	13_43077183_4312376	4.588875609	1.19E-07	4.57E-05
	14_21977748_2200373	2.593806101	0.00077328	0.031256699
	15_53441095_5344470	4.871915769	0.00089426	0.033231789
	17_7243729_7243998	6.204208929	3.70E-07	8.53E-05
	18_55772612_5577498	1.721837909	0.00082829	0.032268947
	18_60135192_6014060	1.391413911	0.0014202	0.045398212
	19_42531815_4258968	4.834311338	0.00012207	0.008035897
	21_58773598_5881390	6.602710539	0.00064847	0.027668113
	22_35231465_3523771	5.832885634	1.88E-08	1.44E-05
	22_54855072_5487838	1.824309797	5.46E-07	0.000104877
	22_54855072_5488951	4.839161678	0.00027845	0.014919497
	22_54855072_5491339	1.704748971	0.00054526	0.024632966
	22_59343961_5934500	1.457782915	0.00024387	0.013431765
	26_10158184_1023752	4.529063028	0.00112639	0.038734231
	26_35338092_3534091	6.51743104	6.88E-40	1.59E-36
	28_29741235_2974978	2.711160766	0.00128718	0.042366504
	29_51170118_5117358	1.847648753	0.00106068	0.037320118
	2_29788651_29798577	3.91328946	0.00157615	0.04778231
	3_114116822_1141227	5.520850516	2.71E-06	0.000445436
	3_34884799_34895626	7.529732871	1.42E-09	1.64E-06
	3_34889318_34895626	4.535668778	2.14E-07	6.17E-05
	3_34985049_35013916	5.667813788	2.54E-05	0.002546592
	3_86771940_86828871	2.573734766	0.00082057	0.032268947
	4_1238642_1253045	4.548108246	0.00075522	0.031071999
	4_25410498_25487125	4.56366314	0.00170396	0.049695353
	5_100556810_1005589	1.115277223	0.00050342	0.02359327
	5_115017045_1150411	1.406516887	0.00024485	0.013431765
	5_115881622_1158850	2.135543138	2.11E-06	0.000374664
	5_15084500_15085511	5.30537512	2.25E-05	0.002354098
	5_47761158_47825812	1.056146394	0.00153148	0.0470471
	5_47804778_47825812	1.295398131	6.53E-06	0.000886744
	7_2828319_2833082	2.941113908	0.00057381	0.024944583
	7_92969600_92969988	1.625068697	0.00011995	0.008035897
	8_99244651_99252010	4.916330718	0.00057259	0.024944583
	8_99251909_99263537	4.957426982	0.00089253	0.033231789
	9_21247249_21260641	4.293042812	0.00014027	0.00828667

	11_45319520_4532586	-4.676639913	0.00010821	0.019401369
	12_71003761_7100608	-2.256114822	4.35E-05	0.009671986
	13_27298457_2730618	-1.899452484	0.00037446	0.039932476
	16_74953105_7495401	-1.357297994	9.95E-06	0.003315525
	17_2211680_2212292	-1.592168987	0.00025281	0.033699099
	18_55779595_5580175	-25.95532797	2.42E-08	3.23E-05
	19_13892761_1389286	-6.064897145	0.00017808	0.026374859
	19_42521928_4255575	-22.07194469	6.19E-07	0.000550114
	1_134405843_1344085	-1.493616317	0.00013683	0.021458722
	21_41938603_4196389	-1.297877216	3.77E-05	0.009146117
	23_23531594_2358109	-37.36245334	3.76E-38	1.00E-34
	29_9003287_9021328	-1.476036772	0.00042376	0.041842511
	3_41133689_41148202	-4.621693239	0.00011644	0.019401369
	3_41133689_41155760	-5.094543478	7.97E-06	0.003035588
	3_42554595_42560714	-4.92492401	0.00037229	0.039932476
	5_87186605_87192046	-3.22707709	0.00050184	0.044596755
W12 vs. W2	10_87268357_8727359	4.87955722	0.00020077	0.028170877
	11_102883877_102885	1.090495869	0.00060352	0.048756933
	11_283882_295588	1.165632201	0.00048255	0.044361567
	13_43077183_4312376	3.725313463	1.38E-05	0.003683358
	15_53441095_5344470	5.668301643	0.00011117	0.019401369
	17_7243729_7243998	4.158777361	0.00036088	0.039932476
	18_40246240_4024724	22.72392348	1.04E-06	0.000695183
	21_58773598_5881390	7.728267133	7.75E-05	0.015903587
	21_67633695_6764312	4.701314664	0.00041859	0.041842511
	22_35231465_3523771	2.032257181	0.00046548	0.044319995
	2_13275369_13275494	4.488235627	0.00034289	0.039932476
	3_27375420_27432684	4.513347452	0.00055514	0.047742204
	5_107949539_1079549	1.939812696	5.41E-06	0.002402038
	5_112048568_1120670	10.68168978	1.38E-05	0.003683358
	5_115017045_1150411	1.847495859	4.31E-06	0.002299793
	6_89006064_89010260	4.622797436	0.00060042	0.048756933
	7_92969600_92969988	1.565919688	0.00034092	0.039932476
	14_45049116_4509150	-1.47938491	7.91E-05	0.025347407
	19_42521928_4255575	-21.77334571	8.76E-07	0.000448869
	23_23531594_2358109	-37.68462056	8.65E-39	2.22E-35
	3_41133689_41148202	-4.848632067	4.43E-05	0.016212368
	3_41133689_41155760	-5.194303961	4.63E-06	0.00197815
	3_41133689_41157200	-2.954967512	0.00021376	0.04980488
W12 vs. W4	18_40246240_4024724	23.0815028	7.04E-07	0.000448869
	21_58773598_5881390	10.54998211	5.29E-07	0.000448869
	25_37565394_3761808	26.33951236	4.83E-25	6.19E-22
	3_86771940_86828871	3.608245823	0.00012503	0.035592116
	5_115881622_1158850	1.542051928	0.00024753	0.04980488
	8_62165931_62171356	1.622612169	0.00025272	0.04980488
	8_94379232_94407339	1.621673879	0.00025214	0.04980488
	16_4481845_4562530	26.62592393	4.45E-11	1.56E-08
	21_58773598_5881390	7.588733343	9.97E-05	0.011666398
	25_2382128_2396954	2.892639771	0.00025236	0.022144742
	27_6986821_7032062	1.826337567	0.00085754	0.047996871
	3_72167775_72194318	1.726369232	0.00078749	0.047996871
	5_112048568_1120670	9.681647686	8.15E-05	0.011449022
W12 vs. W8	5_115017045_1150411	1.27920267	0.00088883	0.047996871
	6_68272249_68299586	1.057595867	0.00021677	0.02173884
	7_94525151_94525616	1.277893661	0.00064905	0.045562995
	18_55779595_5580175	-24.87747882	8.95E-08	2.09E-05

19_42521928_4255575	-21.2758484	1.55E-06	0.000272616
21_58790959_5884165	-6.477169178	0.00053609	0.041814882
23_23531594_2358109	-36.9987137	1.93E-37	1.35E-34

ent in Goats
