# Supplementary material for: Comprehensive analysis of circular RNA expression dynamics and competitive endogenous RNA network mechanisms during postnatal liver development in juvenile goats
Source: Anim Biosci. 2025 Nov 25;39(4):250689. doi: 10.5713/ab.250689 (PMC13064993; doi:10.5713/ab.250689)
Supplement: Supplementary file 5 [file ab-250689-Supplementary-5.pdf]

Supplement 5. Correlation analysis of miRNA, mRNA, and circRNA within the ceRNA regulatory network

ceRNA1 id	ceRNA1 ty	ceRNA2 ty	ceRNA2 gene id	Pvalue	Corr	Corr pvalu	Corr padju
12_504594	circRNA	mRNA	ENSCHIG00000019503	0.035714	0.629736	0.000743	0.00895
12_504594	circRNA	mRNA	ENSCHIG00000013856	0.017857	0.578495	0.002451	0.015488
26_772842	circRNA	mRNA	ENSCHIG00000018825	0.017857	0.726678	3.89E-05	0.001707
26_772842	circRNA	mRNA	ENSCHIG00000025310	0.035714	0.570329	0.002913	0.017085
26_772842	circRNA	mRNA	ENSCHIG00000005553	0.017857	0.629209	0.000753	0.009001
26_772842	circRNA	mRNA	ENSCHIG00000006979	0.017857	0.496154	0.011652	0.03417
26_772842	circRNA	mRNA	ENSCHIG00000013388	0.017857	0.412466	0.040463	0.071679
26_772842	circRNA	mRNA	ENSCHIG00000016424	0.017857	0.7553	1.27E-05	0.001415
26_104557	circRNA	mRNA	ENSCHIG00000013835	0.021661	0.535872	0.005763	0.023213
21_609961	circRNA	mRNA	ENSCHIG00000001082	0.017857	0.549346	0.004451	0.02031
21_609961	circRNA	mRNA	ENSCHIG00000023131	0.017857	0.478337	0.015576	0.03947
13_430771	circRNA	mRNA	ENSCHIG00000015178	0.013636	0.742835	2.11E-05	0.001502
13_430771	circRNA	mRNA	ENSCHIG00000017340	0.013636	0.782458	3.81E-06	0.001155
13_430771	circRNA	mRNA	ENSCHIG00000010157	0.037121	0.471629	0.017309	0.042148
22_548550	circRNA	mRNA	ENSCHIG00000012284	0.006494	0.490472	0.012803	0.035995
22_548550	circRNA	mRNA	ENSCHIG00000023334	0.018398	0.481645	0.014775	0.038056
22_548550	circRNA	mRNA	ENSCHIG00000021304	0.034714	0.418462	0.037359	0.067791
10_491396	circRNA	mRNA	ENSCHIG00000018190	0.035714	0.497979	0.011301	0.033625
10_491396	circRNA	mRNA	ENSCHIG00000024087	0.035714	0.526735	0.006827	0.02574
10_491396	circRNA	mRNA	ENSCHIG00000010529	0.017857	0.487517	0.013437	0.036406
10_491396	circRNA	mRNA	ENSCHIG00000026139	0.035714	0.775236	5.34E-06	0.001194
10_491396	circRNA	mRNA	ENSCHIG00000003330	0.035714	0.549973	0.004397	0.020251
10_491396	circRNA	mRNA	ENSCHIG00000016564	0.035714	0.425179	0.034109	0.063664
10_491396	circRNA	mRNA	ENSCHIG00000012488	0.017857	0.577121	0.002524	0.015811
10_491284	circRNA	mRNA	ENSCHIG00000020615	0.305215	0.373241	0.066104	0.129081
10_491396	circRNA	mRNA	ENSCHIG00000014187	0.017857	0.705438	8.19E-05	0.002851
11_283882	circRNA	mRNA	ENSCHIG00000016497	0.005736	0.550106	0.004386	0.020251
11_283882	circRNA	mRNA	ENSCHIG00000022824	0.005736	0.485467	0.013893	0.036937
11_283882	circRNA	mRNA	ENSCHIG00000013835	0.011255	0.519231	0.007819	0.027764
11_283882	circRNA	mRNA	ENSCHIG00000011661	0.001948	0.573244	0.00274	0.016661
11_283882	circRNA	mRNA	ENSCHIG00000026653	0.001948	0.512999	0.008731	0.028847
11_283882	circRNA	mRNA	ENSCHIG00000021304	0.011255	0.686154	0.000153	0.003984
11_283882	circRNA	mRNA	ENSCHIG00000024517	0.005736	0.488838	0.013151	0.036365
5_1150170	circRNA	mRNA	ENSCHIG00000021304	0.021661	0.572308	0.002795	0.016661
10_150450	circRNA	mRNA	ENSCHIG00000009597	0.035714	0.473532	0.016802	0.041372
10_150450	circRNA	mRNA	ENSCHIG00000011474	0.035714	0.44821	0.024638	0.052463
10_150450	circRNA	mRNA	ENSCHIG00000004184	0.001948	0.579677	0.00239	0.01545
10_150450	circRNA	mRNA	ENSCHIG00000025723	0.035714	0.568072	0.003053	0.017505
10_150450	circRNA	mRNA	ENSCHIG00000020678	0.001948	0.589463	0.00193	0.014461
10_150450	circRNA	mRNA	ENSCHIG00000024279	0.035714	0.595073	0.001702	0.013812
3_4255459	circRNA	mRNA	ENSCHIG00000011185	0.017857	0.456735	0.021725	0.04849
5_9026755	circRNA	mRNA	ENSCHIG00000005553	0.035714	0.400598	0.047206	0.079833
5_9026755	circRNA	mRNA	ENSCHIG00000013388	0.035714	0.538395	0.005496	0.022768
5_9026755	circRNA	mRNA	ENSCHIG00000024123	0.035714	0.669209	0.000254	0.005143
5_9026755	circRNA	mRNA	ENSCHIG00000016424	0.035714	0.493393	0.0122	0.035055
5_9026755	circRNA	mRNA	ENSCHIG00000017740	0.035714	0.475019	0.016415	0.040784
15_534410	circRNA	mRNA	ENSCHIG00000017394	0.035714	0.538833	0.00545	0.0227
24_581889	circRNA	mRNA	ENSCHIG00000000782	0.017857	0.397692	0.048984	0.082042
22_548550	circRNA	mRNA	ENSCHIG00000023334	0.011255	0.507544	0.009601	0.030353
22_548550	circRNA	mRNA	ENSCHIG00000021304	0.021661	0.478462	0.015545	0.039456
LWLT010(	circRNA	mRNA	ENSCHIG00000019503	0.00974	0.577926	0.002481	0.015603
LWLT010(	circRNA	mRNA	ENSCHIG00000019256	0.00974	0.463997	0.01947	0.045439
10_212176	circRNA	mRNA	ENSCHIG00000026998	0.017857	0.537607	0.005578	0.02285
10_212176	circRNA	mRNA	ENSCHIG00000025110	0.017857	0.432493	0.030831	0.060578

10_212176 circRNA	mRNA	ENSCHIG00000024435	0.017857	0.432931	0.030643	0.06036
10_212176 circRNA	mRNA	ENSCHIG00000020615	0.080645	0.563129	0.00338	0.025703
10_212176 circRNA	mRNA	ENSCHIG00000011087	0.017857	0.652224	0.000411	0.006446
3_3498504 circRNA	mRNA	ENSCHIG00000009096	0.018398	0.488007	0.01333	0.036365
3_3498504 circRNA	mRNA	ENSCHIG00000013835	0.034714	0.404022	0.045176	0.077065
3_3498504 circRNA	mRNA	ENSCHIG00000023843	0.006494	0.499722	0.010974	0.033112
10_550456 circRNA	mRNA	ENSCHIG00000013185	0.035714	0.547593	0.004606	0.020678
10_550456 circRNA	mRNA	ENSCHIG00000025448	0.035714	0.527378	0.006747	0.025707
10_550456 circRNA	mRNA	ENSCHIG00000017335	0.035714	0.665382	0.000284	0.00517
10_550456 circRNA	mRNA	ENSCHIG00000025948	0.035714	0.590936	0.001868	0.014236
10_550456 circRNA	mRNA	ENSCHIG00000026057	0.035714	0.421966	0.035634	0.065883
10_550456 circRNA	mRNA	ENSCHIG00000016040	0.035714	0.413115	0.040117	0.071472
5_4776115 circRNA	mRNA	ENSCHIG00000014983	0.005952	0.443421	0.026408	0.054993
5_4776115 circRNA	mRNA	ENSCHIG00000016497	0.005952	0.568956	0.002997	0.017385
5_4776115 circRNA	mRNA	ENSCHIG00000026441	0.035714	0.67392	0.000221	0.004951
5_4776115 circRNA	mRNA	ENSCHIG00000013918	0.035714	0.4495	0.024178	0.052058
5_4776115 circRNA	mRNA	ENSCHIG00000013835	0.020216	0.453077	0.022939	0.050311
5_4776115 circRNA	mRNA	ENSCHIG00000023843	0.035714	0.530871	0.006327	0.024769
5_4776115 circRNA	mRNA	ENSCHIG00000002159	0.005952	0.522023	0.007436	0.026997
5_4776115 circRNA	mRNA	ENSCHIG00000026139	0.035714	0.459231	0.020927	0.047496
5_4776115 circRNA	mRNA	ENSCHIG00000011661	0.035714	0.442734	0.02667	0.055234
5_4776115 circRNA	mRNA	ENSCHIG00000026653	0.035714	0.467553	0.018437	0.044148
2_2978865 circRNA	mRNA	ENSCHIG00000013281	0.035714	0.45441	0.02249	0.049605
2_2978865 circRNA	mRNA	ENSCHIG00000020146	0.035714	0.563906	0.003326	0.018342
2_2978865 circRNA	mRNA	ENSCHIG00000012705	0.035714	0.42117	0.03602	0.066068
2_2978865 circRNA	mRNA	ENSCHIG00000010798	0.035714	0.436728	0.029051	0.058251
2_2978865 circRNA	mRNA	ENSCHIG00000011768	0.035714	0.459796	0.02075	0.04723
2_2978865 circRNA	mRNA	ENSCHIG00000020104	0.035714	0.488424	0.01324	0.036365
2_2978865 circRNA	mRNA	ENSCHIG00000013558	0.035714	0.513851	0.008602	0.028847
2_2978865 circRNA	mRNA	ENSCHIG00000010864	0.035714	0.512188	0.008856	0.029045
2_2978865 circRNA	mRNA	ENSCHIG00000017231	0.035714	0.426026	0.033715	0.063248
2_2978865 circRNA	mRNA	ENSCHIG00000017342	0.035714	0.449496	0.024179	0.052058
2_2978865 circRNA	mRNA	ENSCHIG00000011258	0.035714	0.557542	0.003784	0.01924
2_2978865 circRNA	mRNA	ENSCHIG00000023467	0.035714	0.642515	0.000534	0.007502
14_714472 circRNA	mRNA	ENSCHIG00000018825	0.035714	0.545105	0.004834	0.021174
14_714472 circRNA	mRNA	ENSCHIG00000006979	0.035714	0.61	0.001205	0.011616
14_714472 circRNA	mRNA	ENSCHIG00000000053	0.035714	0.554444	0.004026	0.019647
14_714472 circRNA	mRNA	ENSCHIG00000013388	0.035714	0.431705	0.031171	0.060719
14_714472 circRNA	mRNA	ENSCHIG00000023209	0.035714	0.52664	0.006839	0.02574
14_714472 circRNA	mRNA	ENSCHIG00000022611	0.035714	0.443589	0.026344	0.054993
14_714472 circRNA	mRNA	ENSCHIG00000016424	0.035714	0.465126	0.019137	0.045066
10_101040 circRNA	mRNA	ENSCHIG00000005553	0.035714	0.396767	0.049561	0.082655
10_101040 circRNA	mRNA	ENSCHIG00000024379	0.035714	0.415385	0.038927	0.069932
10_101040 circRNA	mRNA	ENSCHIG00000016424	0.035714	0.448941	0.024376	0.052186
22_548550 circRNA	mRNA	ENSCHIG00000016497	0.027056	0.521091	0.007562	0.027287
22_548550 circRNA	mRNA	ENSCHIG00000026602	0.00974	0.502886	0.0104	0.031815
22_548550 circRNA	mRNA	ENSCHIG00000004184	0.027056	0.743698	2.04E-05	0.001502
22_548550 circRNA	mRNA	ENSCHIG00000023334	0.027056	0.51109	0.009028	0.029271
22_548550 circRNA	mRNA	ENSCHIG00000020678	0.027056	0.48299	0.01446	0.03774
10_491284 circRNA	mRNA	ENSCHIG00000013835	0.034714	0.505203	0.009996	0.031246
10_491284 circRNA	mRNA	ENSCHIG00000025593	0.018398	0.432152	0.030978	0.060639
10_491284 circRNA	mRNA	ENSCHIG00000023851	0.006494	0.431603	0.031215	0.060725
10_491284 circRNA	mRNA	ENSCHIG00000026139	0.006494	0.589982	0.001908	0.014364
10_491284 circRNA	mRNA	ENSCHIG00000003330	0.006494	0.418964	0.037108	0.067414
10_491284 circRNA	mRNA	ENSCHIG00000020906	0.018398	0.695645	0.000113	0.003404
10_491284 circRNA	mRNA	ENSCHIG00000006431	0.034714	0.406656	0.043662	0.075545

28_297412	circRNA	mRNA	ENSCHIG00000012120	0.035714	0.486997	0.013552	0.036451
28_297412	circRNA	mRNA	ENSCHIG00000024745	0.017857	0.459399	0.020874	0.047445
7_9432780	circRNA	mRNA	ENSCHIG00000013281	0.035714	0.448163	0.024655	0.052463
7_9432780	circRNA	mRNA	ENSCHIG00000010100	0.035714	0.406154	0.043947	0.075545
7_9432780	circRNA	mRNA	ENSCHIG00000019928	0.035714	0.465306	0.019085	0.04501
7_9432780	circRNA	mRNA	ENSCHIG00000012705	0.035714	0.429313	0.032223	0.061744
7_9432780	circRNA	mRNA	ENSCHIG00000008851	0.035714	0.426154	0.033656	0.063248
7_9432780	circRNA	mRNA	ENSCHIG00000011768	0.035714	0.75	1.58E-05	0.001415
7_9432780	circRNA	mRNA	ENSCHIG00000020104	0.035714	0.619973	0.000948	0.010164
7_9432780	circRNA	mRNA	ENSCHIG00000011059	0.035714	0.677692	0.000198	0.004736
7_9432780	circRNA	mRNA	ENSCHIG00000008795	0.035714	0.428929	0.032395	0.06179
7_9432780	circRNA	mRNA	ENSCHIG00000010864	0.035714	0.518462	0.007927	0.027833
7_9432780	circRNA	mRNA	ENSCHIG00000011258	0.035714	0.59	0.001907	0.014364
7_9432780	circRNA	mRNA	ENSCHIG00000023467	0.035714	0.506567	0.009765	0.030767
26_101581	circRNA	mRNA	ENSCHIG00000021047	0.035714	0.489421	0.013026	0.036306
14_450821	circRNA	mRNA	ENSCHIG00000002342	0.035714	0.415015	0.039119	0.069932
14_450821	circRNA	mRNA	ENSCHIG00000012284	0.000649	0.460443	0.020548	0.047114
14_450821	circRNA	mRNA	ENSCHIG00000023408	0.035714	0.574744	0.002655	0.016239
14_450821	circRNA	mRNA	ENSCHIG00000000607	0.035714	0.571538	0.00284	0.016842
12_736908	circRNA	mRNA	ENSCHIG00000014529	0.035714	0.513077	0.008719	0.028847
21_584030	circRNA	mRNA	ENSCHIG00000006690	0.017857	0.611972	0.00115	0.011397
21_584030	circRNA	mRNA	ENSCHIG00000025399	0.017857	0.596764	0.001638	0.013684
21_584030	circRNA	mRNA	ENSCHIG00000001815	0.017857	0.624645	0.000844	0.009401
21_584030	circRNA	mRNA	ENSCHIG00000024925	0.017857	0.466176	0.018832	0.044716
21_584030	circRNA	mRNA	ENSCHIG00000017192	0.017857	0.566468	0.003156	0.017836
21_584030	circRNA	mRNA	ENSCHIG00000019462	0.017857	0.540675	0.005263	0.022155
21_584030	circRNA	mRNA	ENSCHIG00000015913	0.017857	0.508062	0.009516	0.030166
3_7478945	circRNA	mRNA	ENSCHIG00000007360	0.017857	0.396983	0.049427	0.082518
17_724372	circRNA	mRNA	ENSCHIG00000018190	0.035714	0.475368	0.016325	0.040643
17_724372	circRNA	mRNA	ENSCHIG00000010529	0.017857	0.460053	0.02067	0.047154
17_724372	circRNA	mRNA	ENSCHIG00000026139	0.035714	0.592783	0.001792	0.014043
17_724372	circRNA	mRNA	ENSCHIG00000003330	0.035714	0.594529	0.001723	0.013891
17_724372	circRNA	mRNA	ENSCHIG00000016564	0.035714	0.710606	6.88E-05	0.002504
17_724372	circRNA	mRNA	ENSCHIG00000012488	0.017857	0.469158	0.017986	0.0432
17_724372	circRNA	mRNA	ENSCHIG00000014187	0.017857	0.402575	0.046026	0.078259
5_1158816	circRNA	mRNA	ENSCHIG00000017650	0.001948	0.459023	0.020993	0.047576
5_1158816	circRNA	mRNA	ENSCHIG00000025360	0.005736	0.516923	0.008147	0.027917
5_1158816	circRNA	mRNA	ENSCHIG00000020906	0.005736	0.674105	0.00022	0.004951
5_1508450	circRNA	mRNA	ENSCHIG00000018463	0.035714	0.421323	0.035945	0.066068
5_1508450	circRNA	mRNA	ENSCHIG00000017031	0.035714	0.574824	0.00265	0.016239
5_1508450	circRNA	mRNA	ENSCHIG00000020256	0.017857	0.56515	0.003243	0.018136
4_2541049	circRNA	mRNA	ENSCHIG00000016748	0.027056	0.567914	0.003063	0.017505
4_2541049	circRNA	mRNA	ENSCHIG00000025201	0.027056	0.617429	0.001008	0.010457
4_2541049	circRNA	mRNA	ENSCHIG00000021051	0.00974	0.474947	0.016433	0.040784
13_430618	circRNA	mRNA	ENSCHIG00000021304	0.011255	0.517361	0.008084	0.027862
16_703324	circRNA	mRNA	ENSCHIG00000023843	0.000649	0.542797	0.005054	0.021622
4_2540781	circRNA	mRNA	ENSCHIG00000026624	0.035714	0.500645	0.010804	0.032725
4_2540781	circRNA	mRNA	ENSCHIG00000011418	0.035714	0.45213	0.023261	0.050805
4_2540781	circRNA	mRNA	ENSCHIG00000012394	0.035714	0.647348	0.000469	0.006994
4_2540781	circRNA	mRNA	ENSCHIG00000021066	0.035714	0.406468	0.043768	0.075545
4_2540781	circRNA	mRNA	ENSCHIG00000013044	0.035714	0.471473	0.017352	0.042148
4_2540781	circRNA	mRNA	ENSCHIG00000001609	0.035714	0.406876	0.043537	0.075419
4_2540781	circRNA	mRNA	ENSCHIG00000020256	0.035714	0.580145	0.002366	0.01542
4_2540781	circRNA	mRNA	ENSCHIG00000021733	0.035714	0.60583	0.001329	0.012247
4_2540781	circRNA	mRNA	ENSCHIG00000016306	0.035714	0.676083	0.000207	0.00485
4_2540781	circRNA	mRNA	ENSCHIG00000024745	0.035714	0.543877	0.00495	0.021353

21_199067	circRNA	mRNA	ENSCHIG00000025146	0.017857	0.689376	0.000138	0.003726
11_102883	circRNA	mRNA	ENSCHIG00000015409	0.035714	0.416154	0.03853	0.069434
11_102883	circRNA	mRNA	ENSCHIG00000025064	0.017857	0.4	0.047568	0.080271
11_102883	circRNA	mRNA	ENSCHIG00000021082	0.017857	0.592308	0.001811	0.014043
5_1005568	circRNA	mRNA	ENSCHIG00000024407	0.017857	0.420769	0.036216	0.066177
16_448184	circRNA	mRNA	ENSCHIG00000025399	0.017857	0.429113	0.032313	0.061744
16_448184	circRNA	mRNA	ENSCHIG00000024925	0.017857	0.433673	0.030326	0.059887
17_130839	circRNA	mRNA	ENSCHIG00000026624	0.035714	0.558462	0.003715	0.01924
17_130839	circRNA	mRNA	ENSCHIG00000001609	0.035714	0.470769	0.017543	0.042394
17_130839	circRNA	mRNA	ENSCHIG00000021089	0.035714	0.692308	0.000126	0.003581
8_9924465	circRNA	mRNA	ENSCHIG00000010100	0.017857	0.662757	0.000306	0.005447
8_9924465	circRNA	mRNA	ENSCHIG00000015588	0.035714	0.598057	0.001591	0.013539
8_9924465	circRNA	mRNA	ENSCHIG00000019928	0.017857	0.732882	3.09E-05	0.001707
8_9924465	circRNA	mRNA	ENSCHIG00000020198	0.017857	0.526297	0.006882	0.02574
8_9924465	circRNA	mRNA	ENSCHIG00000012705	0.017857	0.474555	0.016535	0.040906
8_9924465	circRNA	mRNA	ENSCHIG00000019003	0.017857	0.457133	0.021596	0.04849
8_9924465	circRNA	mRNA	ENSCHIG00000017031	0.035714	0.480551	0.015036	0.038538
8_9924465	circRNA	mRNA	ENSCHIG00000010913	0.035714	0.663714	0.000298	0.005361
8_9924465	circRNA	mRNA	ENSCHIG00000023851	0.035714	0.54529	0.004817	0.021174
8_9924465	circRNA	mRNA	ENSCHIG00000013153	0.035714	0.817198	6.19E-07	0.000323
8_9924465	circRNA	mRNA	ENSCHIG00000008851	0.017857	0.687641	0.000146	0.003868
8_9924465	circRNA	mRNA	ENSCHIG00000020104	0.017857	0.508352	0.009468	0.030076
8_9924465	circRNA	mRNA	ENSCHIG00000017650	0.035714	0.471328	0.017391	0.042158
8_9924465	circRNA	mRNA	ENSCHIG00000008192	0.035714	0.436061	0.029326	0.058613
8_9924465	circRNA	mRNA	ENSCHIG00000025124	0.035714	0.841096	1.40E-07	0.00011
8_9924465	circRNA	mRNA	ENSCHIG00000008795	0.017857	0.691507	0.000129	0.003608
8_9924465	circRNA	mRNA	ENSCHIG00000010864	0.017857	0.51345	0.008662	0.028847
8_9924465	circRNA	mRNA	ENSCHIG00000023467	0.017857	0.58455	0.00215	0.014834
8_9924465	circRNA	mRNA	ENSCHIG00000025997	0.017857	0.498613	0.011181	0.033479
3_8677194	circRNA	mRNA	ENSCHIG00000026691	0.037121	0.646277	0.000483	0.006999
3_8677194	circRNA	mRNA	ENSCHIG00000022376	0.037121	0.480796	0.014978	0.038493
3_8677194	circRNA	mRNA	ENSCHIG00000018949	0.001263	0.62855	0.000766	0.009082
3_8677194	circRNA	mRNA	ENSCHIG00000021109	0.013636	0.456717	0.021731	0.04849
29_900328	circRNA	mRNA	ENSCHIG00000017075	0.017857	0.438931	0.028159	0.057149
29_900328	circRNA	mRNA	ENSCHIG00000019503	0.035714	0.583189	0.002215	0.015081
29_900328	circRNA	mRNA	ENSCHIG00000007274	0.017857	0.478505	0.015535	0.039456
29_900328	circRNA	mRNA	ENSCHIG00000015358	0.017857	0.509231	0.009325	0.029863
29_900328	circRNA	mRNA	ENSCHIG00000016185	0.017857	0.444663	0.025939	0.054451
19_425318	circRNA	mRNA	ENSCHIG00000018463	0.00974	0.453538	0.022783	0.050109

Hit miRNA corr
mir-150:chi-miR-150(-0.58153846
mir-150:chi-miR-150(-0.58153846
mir-483:chi-miR-483(-0.50230769
mir-483:chi-miR-483(-0.50230769
mir-483:chi-miR-483(-0.50230769
mir-483:chi-miR-483(-0.50230769
mir-483:chi-miR-483(-0.50230769
mir-483:chi-miR-483(-0.50230769
mir-483:chi-miR-483(-0.61127140
mir-29:chi-miR-29a-3p(-0.5853846
mir-29:chi-miR-29a-3p(-0.5853846
mir-1343:chi-miR-134(-0.5143296
mir-33:chi-miR-33b-3p(-0.691937
mir-33:chi-miR-33b-3p(-0.691937
mir-33:chi-miR-33b-3p(-0.672181
mir-17:chi-miR-18a-3p(-0.782307
mir-33:chi-miR-33b-3p(-0.672181
mir-493:chi-miR-493-3p(-0.72799
mir-493:chi-miR-493-3p(-0.72799
mir-493:chi-miR-493-3p(-0.72799
mir-493:chi-miR-493-3p(-0.72799
mir-493:chi-miR-493-3p(-0.72799
mir-493:chi-miR-493-3p(-0.72799
mir-493:chi-miR-493-3p(-0.72799
mir-542:chi-miR-542-5p(-0.55144
mir-493:chi-miR-493-3p(-0.72799
mir-17:chi-miR-18a-3p(-0.67|-0.72
mir-432:chi-miR-432-5p(-0.69461
mir-17:chi-miR-18a-3p(-0.67|-0.69
mir-17:chi-miR-18a-3p(-0.67|-0.59
mir-154:chi-miR-382-5p(-0.657692
mir-17:chi-miR-18a-3p(-0.67|-0.63
mir-432:chi-miR-432-5p(-0.69461
mir-17:chi-miR-18a-3p(-0.5415384
mir-188:chi-miR-532-3p(-0.76443
mir-188:chi-miR-532-3p(-0.76443
mir-188:chi-miR-532-3p(-0.76443
mir-188:chi-miR-532-3p(-0.76443
mir-188:chi-miR-532-3p(-0.76443
mir-188:chi-miR-532-3p(-0.76443
mir-708:chi-miR-708-3p(-0.56531
mir-483:chi-miR-483(-0.55245914
mir-483:chi-miR-483(-0.55245914
mir-33:chi-miR-33b-5p(-0.741694
mir-483:chi-miR-483(-0.55245914
mir-483:chi-miR-483(-0.55245914
mir-322:chi-miR-424-5p(-0.53233
mir-3955:chi-miR-3955-5p(-0.603
mir-17:chi-miR-18a-3p(-0.5592307
mir-33:chi-miR-33b-3p(-0.5228934
mir-150:chi-miR-150(-0.66911983
mir-146:chi-miR-146b-5p(-0.6633
mir-542:chi-miR-542-5p(-0.77639
mir-542:chi-miR-542-5p(-0.77639

mir-542:chi-miR-542-5p(-0.776395
mir-542:chi-miR-542-5p(-0.776395
mir-542:chi-miR-542-5p(-0.776395
mir-127:chi-miR-127-5p(-0.633960
mir-17:chi-miR-18a-3p(-0.6290679
mir-412:chi-miR-412-3p(-0.657198
mir-1343:chi-miR-134(-0.60616682
mir-1343:chi-miR-134(-0.60616682
mir-1343:chi-miR-134(-0.60616682
mir-1343:chi-miR-134(-0.60616682
mir-127:chi-miR-127-5p(-0.596147
mir-1343:chi-miR-134(-0.60616682
mir-188:chi-miR-532-3p(-0.615384
mir-188:chi-miR-532-3p(-0.615384
mir-188:chi-miR-532-3p(-0.615384
mir-188:chi-miR-532-3p(-0.615384
mir-432:chi-miR-432-5p(-0.641538
mir-412:chi-miR-412-3p(-0.660769
mir-432:chi-miR-432-5p(-0.641538
mir-432:chi-miR-432-5p(-0.641538
mir-17:chi-miR-18a-3p(-0.5800000
mir-154:chi-miR-382-5p(-0.608461
mir-296:chi-miR-296-3p(-0.588429
mir-197:chi-miR-197-3p(-0.584128
mir-296:chi-miR-296-3p(-0.588429
mir-197:chi-miR-197-3p(-0.584128
mir-296:chi-miR-296-3p(-0.588429
mir-296:chi-miR-296-3p(-0.588429
mir-197:chi-miR-197-3p(-0.584128
mir-296:chi-miR-296-3p(-0.588429
mir-197:chi-miR-197-3p(-0.584128
mir-197:chi-miR-197-3p(-0.584128
mir-296:chi-miR-296-3p(-0.588429
mir-296:chi-miR-296-3p(-0.588429
mir-483:chi-miR-483(-0.52|-0.5066
mir-483:chi-miR-483(-0.52|-0.5046
mir-412:chi-miR-412-3p(-0.511538
mir-483:chi-miR-483(-0.52|-0.5275
mir-483:chi-miR-483(-0.52|-0.6328
mir-412:chi-miR-412-3p(-0.511538
mir-483:chi-miR-483(-0.52|-0.6346
mir-483:chi-miR-483(-0.53076923
mir-34:chi-miR-34c-5p(-0.5215384
mir-483:chi-miR-483(-0.53076923
mir-188:chi-miR-532-3p(-0.615862
mir-1343:chi-miR-134(-0.6576701
mir-188:chi-miR-532-3p(-0.615862
mir-17:chi-miR-18a-3p(-0.6488261
mir-188:chi-miR-532-3p(-0.615862
mir-432:chi-miR-432-5p(-0.655492
mir-296:chi-miR-296-3p(-0.526783
mir-296:chi-miR-296-3p(-0.526783
mir-432:chi-miR-432-5p(-0.655492
mir-493:chi-miR-493-3p(-0.570713
mir-296:chi-miR-296-3p(-0.526783
mir-296:chi-miR-296-3p(-0.526783

mir-130:chi-miR-130b-5p(-0.61685
mir-130:chi-miR-130b-5p(-0.61685
mir-296:chi-miR-296-3p(-0.592307
mir-296:chi-miR-296-3p(-0.592307
mir-296:chi-miR-296-3p(-0.592307
mir-296:chi-miR-296-3p(-0.592307
mir-296:chi-miR-296-3p(-0.592307
mir-296:chi-miR-296-3p(-0.592307
mir-296:chi-miR-296-3p(-0.592307
mir-129:chi-miR-129-3p(-0.526639
mir-296:chi-miR-296-3p(-0.592307
mir-296:chi-miR-296-3p(-0.592307
mir-296:chi-miR-296-3p(-0.592307
mir-296:chi-miR-296-3p(-0.592307
mir-3955:chi-miR-3955-5p(-0.5652
mir-17:chi-miR-18a-3p(-0.5361538
mir-17:chi-miR-18a-3p(-0.5361538
mir-17:chi-miR-18a-3p(-0.5361538
mir-17:chi-miR-18a-3p(-0.5361538
mir-136:chi-miR-136-5p(-0.563846
mir-497:chi-miR-497-5p(-0.577612
mir-497:chi-miR-497-5p(-0.577612
mir-497:chi-miR-497-5p(-0.577612
mir-497:chi-miR-497-5p(-0.577612
mir-497:chi-miR-497-5p(-0.577612
mir-497:chi-miR-497-5p(-0.577612
mir-497:chi-miR-497-5p(-0.577612
mir-365:chi-miR-365-3p(-0.660236
mir-493:chi-miR-493-3p(-0.654344
mir-493:chi-miR-493-3p(-0.654344
mir-493:chi-miR-493-3p(-0.654344
mir-493:chi-miR-493-3p(-0.654344
mir-493:chi-miR-493-3p(-0.654344
mir-493:chi-miR-493-3p(-0.654344
mir-493:chi-miR-493-3p(-0.654344
mir-197:chi-miR-197-3p(-0.500769
mir-197:chi-miR-197-3p(-0.500769
mir-296:chi-miR-296-3p(-0.650769
mir-130:chi-miR-130b-5p(-0.60846
mir-130:chi-miR-130b-5p(-0.60846
mir-130:chi-miR-130b-5p(-0.60846
mir-130:chi-miR-130b-5p(-0.57321
mir-17:chi-miR-18a-3p(-0.5157297
mir-17:chi-miR-18a-3p(-0.5157297
mir-33:chi-miR-33b-3p(-0.6078980
mir-412:chi-miR-412-3p(-0.521538
mir-500:chi-miR-502b-3p(-0.63473
mir-130:chi-miR-130b-5p(-0.57606
mir-500:chi-miR-502b-3p(-0.63473
mir-500:chi-miR-502b-3p(-0.63473
mir-500:chi-miR-502b-3p(-0.63473
mir-500:chi-miR-502b-3p(-0.63473
mir-130:chi-miR-130b-5p(-0.57606
mir-500:chi-miR-502b-3p(-0.63473
mir-500:chi-miR-502b-3p(-0.63473
mir-130:chi-miR-130b-5p(-0.57606

mir-29:chi-miR-29a-5p(-0.5020196
mir-433:chi-miR-433(-0.55923076
mir-433:chi-miR-433(-0.55923076
mir-433:chi-miR-433(-0.55923076
mir-379:chi-miR-758(-0.65538461
mir-497:chi-miR-497-5p(-0.50517
mir-497:chi-miR-497-5p(-0.50517
mir-500:chi-miR-502b-3p(-0.5914
mir-500:chi-miR-502b-3p(-0.5914
mir-500:chi-miR-502b-3p(-0.5914
mir-296:chi-miR-296-3p(-0.62460
mir-296:chi-miR-296-3p(-0.62460
mir-296:chi-miR-296-3p(-0.62460
mir-296:chi-miR-296-3p(-0.62460
mir-296:chi-miR-296-3p(-0.62460
mir-296:chi-miR-296-3p(-0.62460
mir-296:chi-miR-296-3p(-0.62460
mir-296:chi-miR-296-3p(-0.62460
mir-296:chi-miR-296-3p(-0.62460
mir-296:chi-miR-296-3p(-0.62460
mir-296:chi-miR-296-3p(-0.62460
mir-296:chi-miR-296-3p(-0.62460
mir-296:chi-miR-296-3p(-0.62460
mir-296:chi-miR-296-3p(-0.62460
mir-296:chi-miR-296-3p(-0.62460
mir-296:chi-miR-296-3p(-0.62460
mir-296:chi-miR-296-3p(-0.62460
mir-296:chi-miR-296-3p(-0.62460
mir-296:chi-miR-296-3p(-0.62460
mir-296:chi-miR-296-3p(-0.62460
mir-296:chi-miR-296-3p(-0.62460
mir-296:chi-miR-296-3p(-0.62460
mir-433:chi-miR-433(-0.6947770
mir-433:chi-miR-433(-0.6947770
mir-322:chi-miR-424-3p(-0.59351
mir-433:chi-miR-433(-0.6947770
mir-103:chi-miR-103-3p(-0.57|-0.6
mir-103:chi-miR-103-3p(-0.57|-0.6
mir-103:chi-miR-103-3p(-0.57|-0.5
mir-103:chi-miR-103-3p(-0.57|-0.6
mir-103:chi-miR-103-3p(-0.57|-0.5
mir-1343:chi-miR-134(-0.6025303
