# Supplementary material for: Comprehensive analysis of circular RNA expression dynamics and competitive endogenous RNA network mechanisms during postnatal liver development in juvenile goats
Source: Anim Biosci. 2025 Nov 25;39(4):250689. doi: 10.5713/ab.250689 (PMC13064993; doi:10.5713/ab.250689)
Supplement: Supplementary file 6 [file ab-250689-Supplementary-6.pdf]

Supplement 6. KEGG Enrichment Analysis of DEGs in the ceRNA Network

Number	Pathway id	Descriptior	Ratio_in_st	Ratio_in_p	Rich factor	Pvalue	Padjust	First Categ
5	map04672	Intestinal ir	5/92	86/16489	0.05814	0.000118	0.017019	Organismal
5	map00140	Steroid hor	5/92	119/16489	0.042017	0.000537	0.025752	Metabolism
5	map00980	Metabolism	5/92	111/16489	0.045045	0.00039	0.028071	Metabolism
5	map05204	Chemical c	5/92	140/16489	0.035714	0.001118	0.040235	Human Dis
4	map04920	Adipocytok	4/92	100/16489	0.04	0.002366	0.068135	Organismal
4	map00982	Drug metat	4/92	106/16489	0.037736	0.002922	0.070133	Metabolism
2	map00061	Fatty acid t	2/92	16/16489	0.125	0.003512	0.072245	Metabolism
2	map04964	Proximal tu	2/92	32/16489	0.0625	0.0137	0.179351	Organismal
4	map04068	FoxO signa	4/92	170/16489	0.023529	0.015102	0.181219	Environme
4	map04659	Th17 cell d	4/92	151/16489	0.02649	0.010132	0.182375	Organismal
3	map05140	Leishmania	3/92	98/16489	0.030612	0.017463	0.19344	Human Dis
4	map04931	Insulin resi	4/92	165/16489	0.024242	0.01367	0.196846	Human Dis
5	map04020	Calcium sig	5/92	247/16489	0.020243	0.012399	0.198392	Environme
4	map05162	Measles	4/92	217/16489	0.018433	0.03323	0.217505	Human Dis
2	map05340	Primary im	2/92	50/16489	0.04	0.031721	0.217516	Human Dis
7	map04151	PI3K-Akt s	7/92	499/16489	0.014028	0.021486	0.220999	Environme
2	map00790	Folate bios	2/92	45/16489	0.044444	0.026098	0.221069	Metabolism
3	map04662	B cell recep	3/92	109/16489	0.027523	0.023077	0.221536	Organismal
1	map00232	Caffeine m	1/92	8/16489	0.125	0.043783	0.225169	Metabolism
3	map05414	Dilated car	3/92	118/16489	0.025424	0.028316	0.226524	Human Dis
2	map05310	Asthma	2/92	59/16489	0.033898	0.042907	0.228836	Human Dis
2	map00620	Pyruvate m	2/92	64/16489	0.03125	0.049665	0.2307	Metabolism
3	map05323	Rheumatoi	3/92	144/16489	0.020833	0.046676	0.23177	Human Dis
3	map04064	NF-kappa I	3/92	150/16489	0.02	0.051576	0.23209	Environme
5	map04060	Cytokine-c	5/92	299/16489	0.016722	0.025963	0.233665	Environme
3	map04922	Glucagon s	3/92	139/16489	0.021583	0.042779	0.236931	Organismal
4	map04080	Neuroactiv	4/92	247/16489	0.016194	0.049447	0.237343	Environme
3	map03320	PPAR signa	3/92	123/16489	0.02439	0.031477	0.238559	Organismal
3	map04658	Th1 and Th	3/92	123/16489	0.02439	0.031477	0.238559	Organismal
3	map04640	Hematopoi	3/92	134/16489	0.022388	0.039055	0.244516	Organismal
5	map05169	Epstein-Ba	5/92	342/16489	0.01462	0.042483	0.244704	Human Dis
2	map05143	African try	2/92	58/16489	0.034483	0.0416	0.2496	Human Dis

Second Cat	Gene_ids	Gene_name
Immune sy	ENSCHIG(	PIGR;;TNF
Lipid metal	ENSCHIG(	HSD17B2;
Xenobiotic	ENSCHIG(	;CYP1A2;;
Cancer: ov	ENSCHIG(	;CYP1A2;;
Endocrine	ENSCHIG(	PCK1;NFK
Xenobiotic	ENSCHIG(	;CYP1A2;;
Lipid metal	ENSCHIG(	ACACB;
Excretory s	ENSCHIG(	PCK1;
Signal tran	ENSCHIG(	PCK1;KLF
Immune sy	ENSCHIG(	NFKBIA;C
Infectious	ENSCHIG(	NFKBIA;;
Endocrine	ENSCHIG(	PCK1;NFK
Signal tran	ENSCHIG(	SLC8A3;C
Infectious	ENSCHIG(	NFKBIA;H
Immune di	ENSCHIG(	;
Signal tran	ENSCHIG(	PCK1;IL6F
Metabolism	ENSCHIG(	ALPL;FPG
Immune sy	ENSCHIG(	NFKBIA;;
Biosynthes	ENSCHIG(	CYP1A2
Cardiovasc	ENSCHIG(	;;ITGB7
Immune di	ENSCHIG(	;
Carbohydra	ENSCHIG(	PCK1;ACA
Immune di	ENSCHIG(	TNFSF13;;
Signal tran	ENSCHIG(	NFKBIA;;
Signaling n	ENSCHIG(	IL21R;IL6I
Endocrine	ENSCHIG(	PCK1;ACA
Signaling n	ENSCHIG(	S1PR4;CH
Endocrine	ENSCHIG(	PCK1;;CY
Immune sy	ENSCHIG(	NFKBIA;C
Immune sy	ENSCHIG(	IL6R;;
Infectious	ENSCHIG(	NFKBIA;C
Infectious	ENSCHIG(	;
